# Framework for Brain-Derived Dimensions of Psychopathology

**DOI:** 10.1001/jamapsychiatry.2025.1246

**Published:** 2025-06-18

**Authors:** Tristram A. Lett, Nilakshi Vaidya, Tianye Jia, Elli Polemiti, Tobias Banaschewski, Arun L. W. Bokde, Herta Flor, Antoine Grigis, Hugh Garavan, Penny Gowland, Andreas Heinz, Rüdiger Brühl, Jean-Luc Martinot, Marie-Laure Paillère Martinot, Eric Artiges, Frauke Nees, Dimitri Papadopoulos Orfanos, Herve Lemaitre, Tomáš Paus, Luise Poustka, Argyris Stringaris, Lea Waller, Zuo Zhang, Jeanne Winterer, Yuning Zhang, Michael N. Smolka, Robert Whelan, Ulrike Schmidt, Julia Sinclair, Henrik Walter, Jianfeng Feng, Trevor W. Robbins, Sylvane Desrivières, Andre Marquand, Gunter Schumann

**Affiliations:** 1 Centre for Population Neuroscience and Stratified Medicine, Department of Psychiatry and Psychotherapy, Charité Universitätsmedizin, Berlin, Germany; 2 Institute for Science and Technology of Brain-inspired Intelligence, Fudan University, Shanghai, China; 3 Department of Child and Adolescent Psychiatry and Psychotherapy, Central Institute of Mental Health, Medical Faculty Mannheim, Heidelberg University, Mannheim, Germany; 4 Discipline of Psychiatry, School of Medicine and Trinity College Institute of Neuroscience, Trinity College Dublin, Dublin, Ireland; 5 Institute of Cognitive and Clinical Neuroscience, Central Institute of Mental Health, Medical Faculty Mannheim, Heidelberg University, Mannheim, Germany; 6 Department of Psychology, School of Social Sciences, University of Mannheim, Mannheim, Germany; 7 NeuroSpin, Commissariat à l’Energie Atomique, Université Paris-Saclay, Gif-sur-Yvette, France; 8 Department of Psychiatry and Psychology, University of Vermont, Burlington; 9 Sir Peter Mansfield Imaging Centre School of Physics and Astronomy, University of Nottingham, University Park, Nottingham, United Kingdom; 10 Department of Psychiatry and Psychotherapy, Charité Campus Mitte, Humboldt-Universität zu Berlin, and Berlin Institute of Health, Berlin, Germany; 11 Physikalisch-Technische Bundesanstalt, Braunschweig and Berlin, Germany; 12 Institut National de la Santé et de la Recherche Médicale, Institut national de la santé et de la recherche médicale U1299 Trajectoires développementales en psychiatrie, Université Paris-Saclay, Ecole Normale supérieure Paris-Saclay, Centre national de la recherche scientifique Unité Mixte de Recherche 9010, Centre Borelli, Gif-sur-Yvette, France; 13 Assistance Publique-Hôpitaux de Paris, Sorbonne Université, Department of Child and Adolescent Psychiatry, Pitié-Salpêtrière Hospital, Paris, France; 14 Institut National de la Santé et de la Recherche Médicale, Université Paris-Saclay Ecole Normale Supérieure Paris-Saclay, Centre national de la recherche scientifique Centre Borelli, Gif-sur-Yvette, France; 15 Psychiatry Department, Établissement Public de Santé Barthélémy Durand, Etampes, France; 16 Institute of Medical Psychology and Medical Sociology, University Medical Center Schleswig-Holstein, Kiel University, Kiel, Germany; 17 Institut des Maladies Neurodégénératives, Unité Mixte de Recherche 5293, Centre national de la recherche scientifique, Commissariat à l’Energie Atomique, Université de Bordeaux, Bordeaux, France; 18 Department of Psychiatry, Faculty of Medicine and Centre Hospitalier Universitaire Sainte-Justine, University of Montreal, Montreal, Quebec, Canada; 19 Department of Psychiatry, University of Toronto, Toronto, Ontario, Canada; 20 Department of Psychiatry and Psychology, University of Toronto, Toronto, Ontario, Canada; 21 Department of Child and Adolescent Psychiatry, Center for Psychosocial Medicine, University Hospital Heidelberg, Heidelberg, Germany; 22 Division of Psychiatry and Department of Clinical, Educational & Health Psychology, University College London, London, United Kingdom; 23 Social, Genetic and Developmental Psychiatry Centre, Institute of Psychiatry, Psychology & Neuroscience, King’s College London, London, United Kingdom; 24 Department of Education and Psychology, Freie Universität Berlin, Berlin, Germany; 25 Psychology Department, University of Southampton, Southampton, United Kingdom; 26 Department of Psychiatry and Psychotherapy, Technische Universität Dresden, Dresden, Germany; 27 School of Psychology and Global Brain Health Institute, Trinity College Dublin, Dublin, Ireland; 28 Department of Psychological Medicine, Centre for Research in Eating and Weight Disorders, Institute of Psychiatry, Psychology and Neuroscience, King’s College London, London, United Kingdom; 29 South London and Maudsley National Health Service Foundation Trust, London, United Kingdom; 30 Clinical and Experimental Sciences, Faculty of Medicine, University of Southampton, Southampton, United Kingdom; 31 Department of Psychology and Behavioural and Clinical Neuroscience Institute, University of Cambridge, Cambridge, United Kingdom; 32 Donders Institute/Radboud University Medical Center, Nijmegen, the Netherlands; 33 Centre for Population Neuroscience and Precision Medicine, National Center for Neurological Disorders, Huashan Hospital, Fudan University, Shanghai, China; 34 Department of Psychiatry and Neuroscience, University of Cambridge, Cambridge, United Kingdom

## Abstract

**Importance:**

Psychiatric diagnoses are not defined by neurobiological measures hindering the development of therapies targeting mechanisms underlying mental illness. Research confined to diagnostic boundaries yields heterogeneous biological results, whereas transdiagnostic studies often investigate individual symptoms in isolation.

**Objective:**

To develop a framework that groups clinical symptoms compatible with *ICD-10* and *DSM-5* according to their covariation and shared brain mechanisms.

**Design, Setting, and Participants:**

This diagnostic study was conducted in 2 samples, the population-based Reinforcement-Related Behaviour in Normal Brain Function and Psychopathology (IMAGEN) cohort (longitudinal assessments at 14, 19, and 23 years; study duration from March 2010 to the present) and the cross-diagnostic Brain Network Based Stratification of Mental Illness (STRATIFY)/Earlier Detection and Stratification of Eating Disorders and Comorbid Mental Illnesses (ESTRA) samples (study duration from October 2016 to September 2023). The samples are from 8 clinical research hospitals in Germany, the UK, France, and Ireland. For the population-based IMAGEN study, 794 of 1253 23-year-old participants had complete assessments including complete clinical assessments and neuroimaging data across all time points. For the cross-diagnostic STRATIFY/ESTRA samples, 209 of 485 participants aged 18 to 26 years had complete clinical and neuroimaging data. The sample included healthy control individuals and patients with alcohol use disorder, major depressive disorder, anorexia nervosa, and bulimia nervosa.

**Exposures:**

Sparse generalized canonical correlation analysis was used to integrate diverse data from clinical symptoms and 7 brain imaging modalities.

**Main Outcomes and Measures:**

The prediction of symptom features was the main outcome. The model was developed in the training set from the IMAGEN Study at age 23 years (70%), then applied in the remaining holdout test sample (30%), the independent STRATIFY/ESTRA patient sample, and longitudinally in the IMAGEN set.

**Results:**

In total, 1003 participants were included (425 male and 578 female; mean [SD] age, 22.1 [1.5] years). The reassembly of existing *ICD-10* and *DSM-5* symptoms revealed 6 cross-diagnostic psychopathology scores. They were consistently associated with multimodal neuroimaging components: excitability and impulsivity (training set: *r*, 0.26; 95% CI, 0.18-0.33; test set: *r*, 0.22; 95% CI, 0.10-0.35; STRATIFY/ESTRA set: *r*, 0.19; 95% CI, 0.07-0.31), depressive mood and distress (training: *r*, 0.30; 95% CI, 0.20-0.38; test: *r*, 0.22; 95% CI, 0.09-0.35; STRATIFY/ESTRA: *r*, 0.19; 95% CI, 0.04-0.33), emotional and behavioral dysregulation (training: *r*, 0.40; 95% CI, 0.31-0.48; test: *r*, 0.17; 95% CI, 0.14-0.36; STRATIFY/ESTRA: *r*, 0.19; 95% CI, 0.06-0.30), stress pathology (training: *r*, 0.32; 95% CI, 0.19-0.43; test: *r*, 0.14; 95% CI, 0.05-0.23; STRATIFY/ESTRA: *r*, 0.12; 95% CI, 0.01-0.22), eating pathology (training: *r*, 0.34; 95% CI, 0.25-0.42; test: *r*, 0.26; 95% CI, 0.15-0.37; STRATIFY/ESTRA: *r*, 0.15; 95% CI, 0.12-0.34), and social fear and avoidance symptoms (training: *r*, 0.31; 95% CI, 0.25-0.42; test: *r*, 0.18; 95% CI, 0.15-0.35; STRATIFY/ESTRA: *r*, 0.12; 95% CI, 0.12-0.33).

**Conclusion and Relevance:**

In this study, the identification of symptom groups of mental illness robustly defined by precisely characterized brain mechanisms enabled the characterization of dimensions of psychopathology based on quantifiable neurobiological measures.

## Key Points

**Question:**

Can existing psychiatric assessments be enhanced by multimodal brain neuroimaging to create neurobiological dimensions of psychopathology?

**Findings:**

In this diagnostic study including 1003 participants, 6 psychopathology scores derived from *ICD-10* and *DSM-5* clinical symptoms were identified that are defined by shared brain mechanisms characterized by brain structure, function, and connectivity.

**Meaning:**

Identifying symptom groups that are specifically associated with quantifiable neurobiological measures may enable the development of precise interventions that target biological mechanisms of psychiatric disorders and allow for quantitative assessment of comorbidity.

## Teaser

This diagnostic study offers a framework for grouping clinical symptoms according to covariation and underlying brain mechanisms.

## Introduction

There has been a growing imperative within psychiatric neuroscience to uncover the biological mechanisms underlying mental health and disease to develop more effective treatments.^1^ A major challenge lies in the classification of psychiatric disorders since their categorization does not follow biological mechanisms. Biological links distinguishing diagnostic criteria, including brain structure,^2^ function,^3^ and connectivity,^4^ are limited, pointing to shared neurobiological substrates across mental illnesses. Dysfunctions within 1 mechanism affect the clinical presentation of more than 1 diagnosis, giving rise to comorbidity.^5^ For a more nuanced understanding of psychiatric symptoms to be achieved, objective means of patient stratification and identification of robust psychiatric biomarkers are needed.

This need is perhaps most apparent in the efforts of biology-driven initiatives, like the National Institute of Mental Health’s Research Domain Criteria framework.^1^ This framework aims to provide data about biological and behavioral processes related to mental health and mental illness. It is not designed to categorize psychiatric disorders. The Hierarchical Taxonomy of Psychopathology maintains a clinical characterization applying clinical spectra and hierarchy.^6^ Hierarchical Taxonomy of Psychopathology constructs are not driven by the biology underlying psychiatric liability. A unifying framework that considers the complex biological variation and the clinical variation concurrently to characterize nosology is warranted.

A potential solution to this challenge is to use existing clinical measures to optimize the link between symptoms and biology, which may lead to the discovery of novel biomarkers and targets for treatment development. We used a data-driven strategy to integrate information from multiple domains, including clinical symptoms, brain structure (white matter fractional anisotropy, cortical thickness, and surface area), as well as intrinsic (resting-state functional magnetic resonance imaging [MRI]) and extrinsic (task functional MRI) brain function. Our model integrates distinct, multimodal neuroimaging features, revealing their linear associations with shared psychiatric symptoms across different disorders. We characterized in a single statistical model a wide variety of psychiatric symptoms and their covariance with a comprehensive multimodal characterization of the brain and established the reproducibility of our model by validating it in 2 samples with similar clinical and neuroimaging assessments: the population-based Reinforcement-Related Behaviour in Normal Brain Function and Psychopathology (IMAGEN) study (longitudinal assessments at 14, 19, and 23 years; study duration from March 2010 to the present) and its clinical follow-up study, Brain Network Based Stratification of Mental Illness (STRATIFY)/Earlier Detection and Stratification of Eating Disorders and Comorbid Mental Illnesses (ESTRA) (study duration from October 2016 to September 2023). Our analysis aims at a novel framework that combines clinical usefulness with biological validity by harnessing current clinical assessments and quantifiable neurobiological measures, such as comprehensive functional and structural neuroimaging data.

## Methods

### Study Design and Participants

In the population-based IMAGEN-cohort, neuroimaging assessments were conducted at ages 14, 19, and 23 years with an additional psychological assessment at 16 years.^7^ In the cross-disorder STRATIFY/ESTRA clinical sample of patients with major depressive disorder, alcohol use disorder, anorexia nervosa, bulimia nervosa, and healthy control individuals, assessments were similar to those of IMAGEN participants at age 23 years. All studies received ethical approval and obtained written informed consent (for detailed information, see eMethods in Supplement 1). Our training model was established in IMAGEN participants at age 23 years and applied to the test sample, earlier IMAGEN neuroimaging assessments, and the STRATIFY/ESTRA sample. All participants self-reported as Western European. Details of the cohorts are available in the eMethods in Supplement 1. Sample sizes are lower than initial recruitment since we only analyzed participants with complete clinical assessments of the Development and Well-Being Assessment (DAWBA)^8^ Strengths and Difficulties Questionnaire (SDQ),^9^ Alcohol Use Disorders Identification Test (AUDIT),^10^ and neuroimaging data including T1-weighted structural MRI, diffusion weighted images, and resting-state and task-based functional MRI.

### Neuroimaging Procedures

MRI acquisition and processing were performed according to IMAGEN guidelines. Details are available in the eMethods in Supplement 1.

### Statistical Analysis

**Figure 1.**
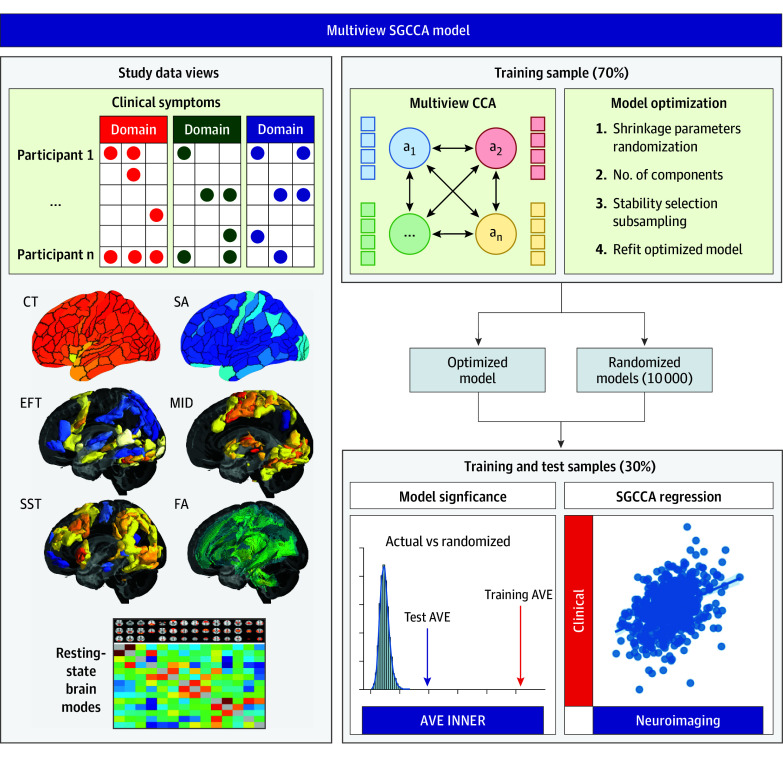
Development of the Sparse Generalized Canonical Correlation Analysis (SGCCA) Model in the Reinforcement-Related Behaviour in Normal Brain Function and Psychopathology (IMAGEN) Study The SGCCA model incorporates 8 distinct datasets (called data views), consisting of both clinical assessments and neuroimaging modalities, from the IMAGEN study. This model is built using 70% of the participants as the training dataset, while the remaining 30% form the test group. The method used is canonical correlation analysis, which uses cross-covariance matrices of 2 or more sets of data views to identify linear combinations (or components) that have maximal correlation. The training data serve several crucial purposes: first, optimizing the model’s parameters, including shrinkage parameters (sparsity); second, determining the suitable number of components; and lastly, performing stability selection (for details, refer to the eMethods in Supplement 1). After establishing the optimal model parameters, the training data are refitted accordingly. Furthermore, 10 000 randomized models are generated by permuting participants among each training data view. This allows us to evaluate the significance of the model within both the training and test datasets for each component. In the training data, the inner average variance explained (AVE) of the actual model is ranked and compared to the inner AVE of the randomized models. Similarly, the test data are fitted to both the actual and randomized models, and their inner AVEs are compared. Last, regression of the data view components is conducted, with clinical component scores as dependent variables and neuroimaging scores as independent variables. This entire process is repeated in both the training and test samples for each of the significant components. CCA indicates canonical correlation analysis; CT, cortical thickness; EFT, emotional face task; FA, fractional anisotropy; MID, monetary incentive delay task; SA, surface area; SST, stop-signal task.

Details of the sparse generalized canonical correlation analysis (SGCCA) model and its derivation have been described elsewhere.^11,12^ Sparse canonical correlation analysis is common among neuroimaging analyzes.^13,14,15^ Prior to inclusion into the SGCCA model, each data view was corrected for age at time of MRI scan, sex, and site. Details of model parameters, optimization, and application are available in the eMethods in Supplement 1. SGCCA uses cross-covariance matrices of 2 or more sets of vectors (or data views) to find the linear combinations (or components) of these data views (clinical or neuroimaging data) that have maximum correlation with each other using gradient descent. We included all data views in a unified model, thus describing multimodal functional, structural, and diffusion MRI relationships in the context of cross-disorder symptom scores (Figure 1).

## Results

The analysis was carried out among 794 participants (366 male and 428 female; aged 23 years) from the IMAGEN cohort and 209 participants (59 male and 150 female; mean [SD] age, 22.1 [1.5] years) from the STRATIFY/ESTRA cohort. We established an optimized SGCCA model (Figure 1), reducing the number of collinear variables in our data views while maximizing the variance explained. The optimal L_1_ sparsity for all data views was λ = 0.3 after 1000 permutations at each of the 10 steps (*z*, 12.6) (eFigure 3A in Supplement 1). We selected 10 components as the point at which the cumulative average variance explained of the full model levels off at 40.4% (eFigure 3B in Supplement 1). The stability selection was performed by randomly selecting 50% of the training data without replacement 10 000 times and retaining the clinical items, brain regions, and resting-state brain mode connectivity variables that appeared in 90% of the subsampled SGCCA models (eFigure 3C and D in Supplement 1). The final model (selected variables, 10 components, and λ_1_ = 1.0) explained 52.7% of the variance among all data views (eFigure 3E in Supplement 1).

**Figure 2.**
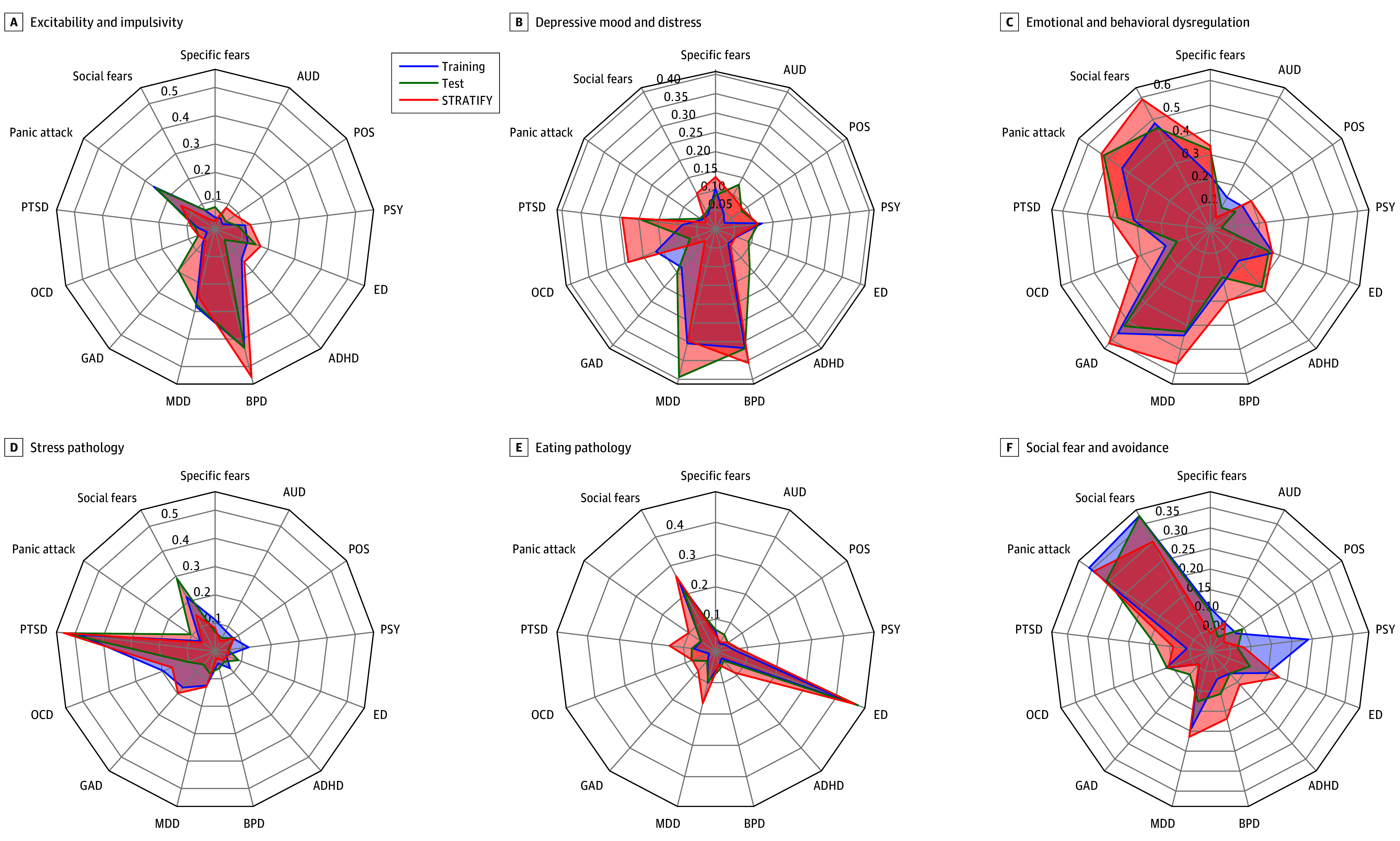
Aggregated Loadings for the Development and Well-Being Assessment (DAWBA) Sections and Alcohol Use Disorders Identification Test (AUDIT) Questionnaire The aggregate loading (radial axis) provides an overview of the association between the symptom scores and the clinical items for the Reinforcement-Related Behaviour in Normal Brain Function and Psychopathology (IMAGEN) training and test samples and the Brain Network Based Stratification of Mental Illness (STRATIFY)/Earlier Detection and Stratification of Eating Disorders and Comorbid Mental Illnesses (ESTRA) sample. The aggregated loading represents the mean of the AUDIT and DAWBA sections for the correlation between the psychopathology scores on the original clinical items. ADHD indicates attention-deficit/hyperactivity disorder; AUD, alcohol use disorder; BPD, bipolar disorder; ED, eating disorders; GAD, generalized anxiety disorder; MDD, major depressive disorder; OCD, obsessive-compulsive disorder; POS, positivity, PSY, psychosis; PTSD, posttraumatic stress disorder.

**Figure 3.**
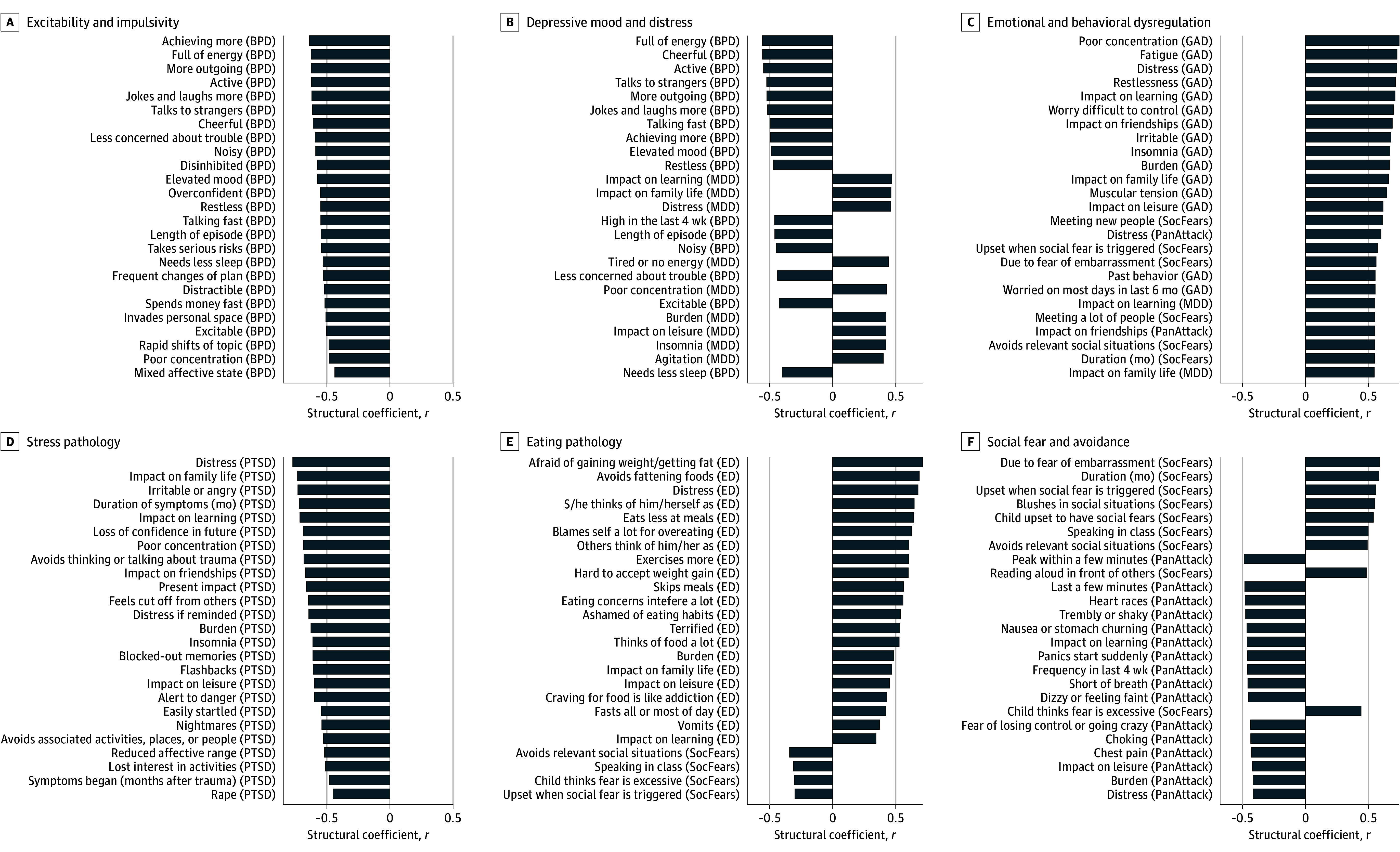
Clinical Contribution to Each Psychopathology Component The top 25 clinical structural coefficients are plotted according to their absolute value for each psychopathology component. The question from the clinical battery is on the y-axis with its corresponding section in parentheses, and the structural coefficients are on the x-axis. All items are significant (false discovery rate–corrected *P* < .05) after 10 000 bootstraps. ADHD indicates attention-deficit/hyperactivity disorder; AUDIT, alcohol use disorders identification test; BPD, bipolar disorder; ED, eating disorder; GAD, general anxiety disorder; MDD, major depressive disorder. OCD, obsessive compulsive disorder; PanAttack, panic attack; PTSD, posttraumatic stress disorder; SDQ, Strength and Difficulties Questionnaire; SocFear, social fears.

In the training data, 10 canonical components we investigated were significant (*z*, 4.4 to 31.9; inner average variance explained, 0.025 to 0.037; permuted *P* < 1.0 × 10^−4^) (eFigure 3F in Supplement 1). In the test data, the first 6 models remained significant (*z*, 1.8 to 10.3; inner average variance, 0.008 to 0.017; permuted *P* = .048 to <1.0 × 10^−4^) (eFigure 3F in Supplement 1). Since the 6 components were significant for the model’s inner canonical correlation, we consider these to be components of interest. For an overview of the contribution of the composition of individual clinical items to psychopathology scores for the 6 components of interest, we calculated the mean DAWBA clinical subdomains, the AUDIT, and SDQ subscales for the structural coefficients (correlation between each psychopathology score and clinical items). The training, test, and STRATIFY/ESTRA samples were similar in terms of psychopathology (Figure 2). Based on these values, we categorized the psychopathology scores as excitability and impulsivity, depressive mood and distress, emotional and behavioral dysregulation, stress pathology, eating pathology, and social fear and avoidance symptoms of components 1 to 6, respectively (Figure 3).

**Figure 4.**
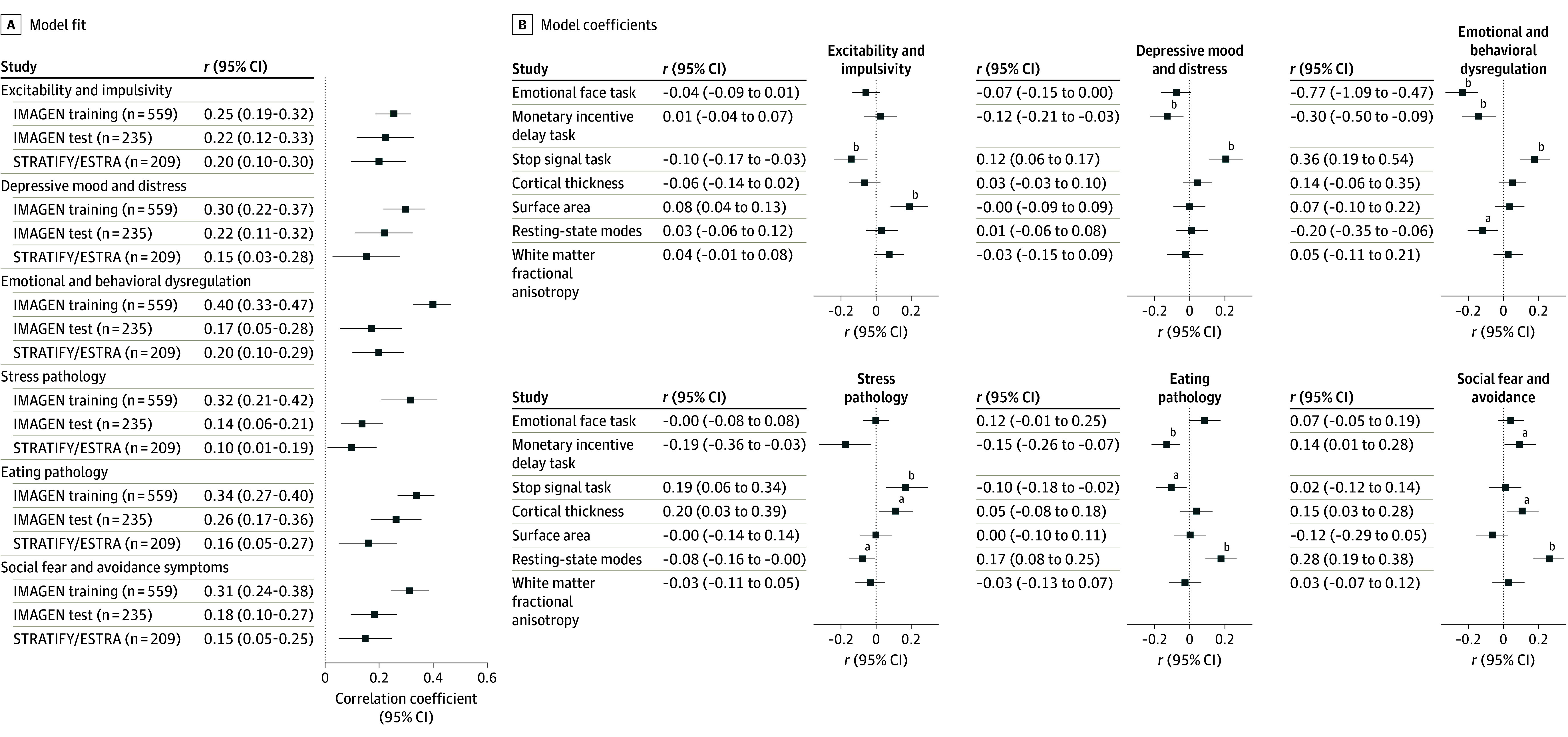
Contribution of Neuroimaging Features to the Psychopathology Symptoms for Each Significant Component Sparse Generalized Canonical Correlation Analysis (SGCCA)–regression of the variates for the training, test Reinforcement-Related Behaviour in Normal Brain Function and Psychopathology (IMAGEN) samples, and Brain Network Based Stratification of Mental Illness (STRATIFY)/Earlier Detection and Stratification of Eating Disorders and Comorbid Mental Illnesses (ESTRA) sample with the psychopathology scores as the response variable and the neuroimaging predictor scores for: the emotional face task, the monetary incentive delay task, the stop-signal task, cortical thickness, surface area, the resting-state brain modes connectivity, and white matter fractional anisotropy. A, Regression model fit and 95% CIs with variate psychopathology scores as the response variable and the neuroimaging predictor scores for the emotional face task, the monetary incentive delay task, the stop-signal task, cortical thickness, surface area, the resting-state brain modes connectivity, and white matter fractional anisotropy. B, Bar charts of the model coefficients. Each regression model underwent 10 000 bootstraps to determine the confidence interval and significance. The horizontal line represents the 95% CI. ^a^Bootstrapped *P* < .05. ^b^Bootstrapped *P* < .008.

Using SGCCA regression to evaluate which neuroimaging scores were contributing to psychopathology scores, we found that each of the 6 symptom component scores predicted their corresponding neuroimaging components scores in the training, test, and STRATIFY/ESTRA samples (Figure 4A) except for the stress pathology, which was only nominally associated in the STRATIFY/ESTRA sample (*r*, 0.10; 95% CI, 0.01-0.19; bootstrapped *P* = .06). The canonical correlations were moderate to low for excitability and impulsivity (training set: *r*, 0.26; 95% CI, 0.18-0.33; *P* < .001; test set: *r*, 0.22; 95% CI, 0.10-0.35; *P* = .002; STRATIFY/ESTRA set: *r*, 0.19; 95% CI, 0.07-0.31; *P* = .002), depressive mood and distress (training set: *r*, 0.30; 95% CI, 0.20-0.38; *P* < .001; test set: *r*, 0.22; 95% CI, 0.09-0.35; *P* < .004; STRATIFY/ESTRA set: *r*, 0.19; 95% CI, 0.04-0.33; *P* = .002), emotional and behavioral dysregulation (training set: *r*, 0.40; 95% CI, 0.31-0.48; *P* < .001; test set: *r*, 0.17; 95% CI, 0.14-0.36; *P* = .003; STRATIFY/ESTRA set: *r*, 0.19; 95% CI, 0.06-0.30; *P* = .001), stress pathology (training set: *r*, 0.32; 95% CI, 0.19-0.43; *P* < .001; test set: *r*, 0.14; 95% CI, 0.05-0.23; *P* = .004; STRATIFY/ESTRA set: *r*, 0.12; 95% CI, 0.01-0.22; *P* = .02), eating pathology (training set: *r*, 0.34; 95% CI, 0.25-0.42; *P* < .001; test set: *r*, 0.26; 95% CI, 0.15-0.37; *P* < .001; STRATIFY/ESTRA set: *r*, 0.15; 95% CI, 0.12-0.34; *P* = .008), and social fear and avoidance symptoms (training set: *r*, 0.31; 95% CI, 0.25-0.42; *P* < .001; test set: *r*, 0.18; 95% CI, 0.15-0.35; *P* < .001; STRATIFY/ESTRA set: *r*, 0.12; 95% CI, 0.12-0.33; *P* = .002). This relationship was generally consistent in the IMAGEN sample at age 14 and age 19 (eFigure 4 in Supplement 1). Replicating the association in the test and STRATIFY/ESTRA data validates the prediction of clinical variates by the neuroimaging variates, since this data constitutes only independent, transformed scores from the SGCCA model.

Next, we asked which variables were driving the association between psychopathology scores and neuroimaging modality scores. We identified which coefficients (neuroimaging modality scores) were significantly associated with the psychopathology scores (bootstrapped *P* < .008) (Figure 4B). Our SGCCA model is not limited to positive covariance since that would assume a priori that the direction of our neuroimaging values is better or worse clinically. Therefore, careful interpretation is needed since the coefficient’s direction can be negative or positive. The significant neuroimaging modality scores are correlated back to their corresponding data. This step is important for identifying which clinical items are most linked to brain regions—information that could be used to develop a parsimonious model to be applied in a clinical setting.

**Figure 5.**
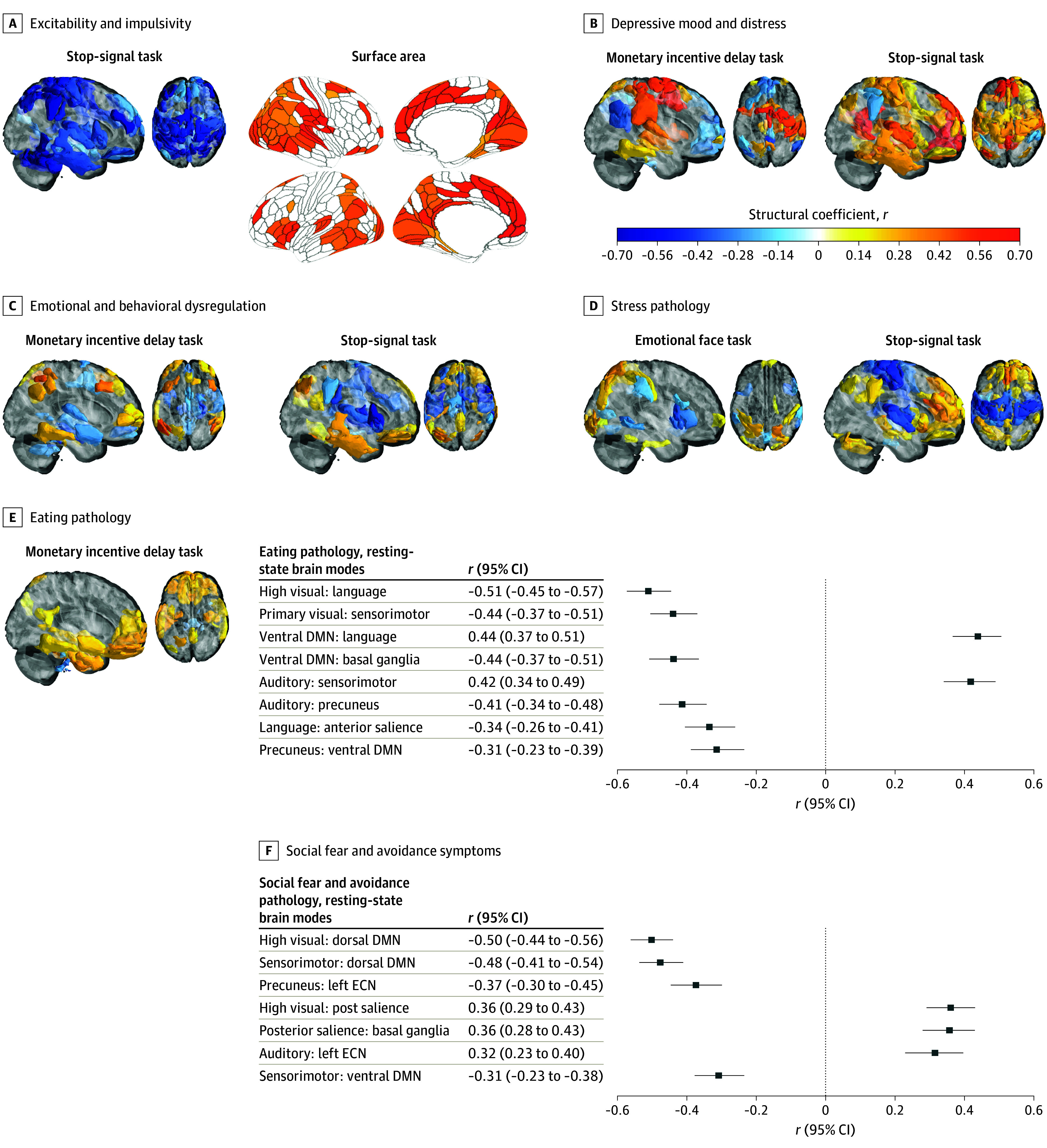
Neuroimaging Loadings for Each Psychopathology Score Significant loadings (structural coefficient *r*) for each score are shown using 10 000 bootstraps and after accounting for false discovery rate –adjusted *P* < .05. Colors ranging from red to dark blue denote significant positive and negative *r* values, respectively. The psychopathology components of interest were excitability and impulsivity (stop-signal task and surface area), depressive mood and distress (monetary incentive delay task and stop-signal task), emotional and behavioral dysregulation (emotional face task, stop-signal task, and monetary incentive delay task), stress pathology (monetary incentive delay task), eating pathology (monetary incentive delay task and resting-state functional magnetic resonance imaging [fMRI] brain modes), and social fear and avoidance (resting-state fMRI brain modes). DMN indicates default mode network; ECN, executive control network.

The psychopathological variables contributing to the excitability and impulsivity symptoms were primarily negatively associated with DAWBA items from the bipolar disorder section (Figure 3). The stop-signal task (SST) negatively correlated in areas involved in frontoparietal executive function which mirrored the surface area correlation in the dorsolateral-prefrontal cortex, anterior-cingulate, and inferior-parietal cortex (Figure 5).

The depressive mood and distress score was correlated with DAWBA items in opposing directions for the bipolar and major depressive disorder sections. The score was negatively correlated with bipolar items related to full of energy, more active, elevated mood, and positively correlated with depressive items, such as miserable daily, impact of depression, tired or low energy, feelings of worthless guilt (Figure 3). Since the excitability and impulsive symptoms score is orthogonal to this score, we consider depressive features to be the defining feature of this component. The monetary incentive delay (MID) and SST scores both correlated with anterior and posterior-cingulate cortex activation but in opposing directions (Figure 5).

The emotional and behavioral dysregulation score was positively correlated with questions related to poor concentration, impact on learning, and distress from the general anxiety disorder DAWBA section with additional correlations from social fears, depression, and panic attack items (Figure 3). The insula and superior temporal gyrus were contributing to the emotional face task (EFT), MID, and SST with the latter correlating with medial-prefrontal cortex activation. The task loadings would suggest a common involvement of salience and ventral attention networks (Figure 5).

The stress pathology score was primarily negatively correlated with items from the posttraumatic stress disorder DAWBA questions (Figure 3). The SST score was correlated with activation in the dorsal anterior-cingulate cortex, insula, as well as the precentral and postcentral gyrus, suggesting an involvement with default mode and salience networks (Figure 5). The cortical thickness score was correlated with thickness in the anterior and posterior cingulate, orbitofrontal, dorsolateral prefrontal, and insular cortices (Figure 5).

The eating pathology score correlated with DAWBA items related to bulimia nervosa rather than anorexia nervosa, although both were present (Figure 3). The MID score was correlated with activation in the striatum, medial-prefrontal cortex, and medial-temporal cortex, suggesting an involvement of limbic and anterior salience networks (Figure 5). The resting-state brain modes score was correlated with a negative relationship between the high-visual and language networks (medial temporal) and a positive relationship between the language network and the ventral default mode network (Figure 5).

The clinical contribution for the social fear and avoidance score were split among social anxiety and panic attack items, indicating a specificity for social anxiety that is differentiated from physical panic symptoms. The resting-state brain modes score correlated with negative relationship among the dorsal default mode network and both the visual and sensorimotor networks in the resting-state brain modes (Figure 5).

## Discussion

In this diagnostic study, we have developed a framework to characterize dimensions of psychopathology based on neurobiological measures. By constructing 6 symptom groups according to covariance and shared structural and functional neuroimaging features across 7 modalities, we have provided mechanistic characterization and identified possible targets for therapeutic intervention. The neuroimaging correlates identified are specific to their symptom group, thus providing precise biomarkers and intervention targets. As our clinical characterization contains *ICD-10* and *DSM-5* symptoms that were reassembled in a manner informed by their shared underlying biology, we preserved the clinical experience accumulated in existing psychopathological characterizations while optimizing them for neurobiological prediction.

The ability to link major psychiatric symptom groups to distinct neuroimaging modalities helps with biological understanding by providing quantifiable measures that are specific to each symptom group. Each component has biological characteristics that are independent of the other components. The model demonstrates predictive stability by replicating in both test and cross-disorder STRATIFY/ESTRA samples. While the model was developed among the IMAGEN participants at age 23 years, the clinical associations largely remained consistent at ages 14 and 19 years, suggesting that the neuroimaging variables may serve as early markers of severe symptoms.

The neuroimaging characterization of psychopathology provides new insight into multimodal brain associations and their symptom-specific liability regions. Previous neuroimaging-CCA studies have identified between 1 and 3 significant components.^5,13,14,15,16^ Our study describes 6 components that capture a wider range of the clinical continuum. The model weights neuroimaging features to provide a relative importance of the anatomical areas to the psychopathology scores. The neuroimaging features are derived from 7 different neuroimaging modalities, which is unique among psychiatric neuroimaging studies that typically focus on a single neuroimaging modality. Therefore, the putative biomarkers we identified are more comprehensive in describing the neurobiology of psychopathology.

The excitability and impulsive symptom score (component 1) was associated with a novel structure-function association involving activation during the SST and surface area in overlapping regions, including the inferior/medial-frontal gyrus, insula, inferior-parietal cortex, caudate, and putamen. These regions play an important role in cognitive control, attention, and response inhibition and are altered in patients with bipolar disorder and their relatives.^17,18^ By demonstrating the contingency of functional activation of these brain areas implicated in behavioral inhibition^19^ on the regional surface area, our finding provides a more refined understanding of the biomarkers that could contribute to impaired inhibitory control.^20^

For the depressive mood and distress score (component 2), we identified a fronto-limbic brain network involved in top-down control and emotion integration^21^ that consists of overlapping activations during SST and MID tasks in the dorsolateral/medial-prefrontal cortex, posterior-cingulate cortex, precuneus, and limbic regions, including the hippocampus and amygdala. This network is specific to depressive symptoms and distinct from component 1.

The emotional and behavioral dysregulation score (component 3) was the only component broadly associated with multiple diagnostic categories with items primarily related to anxiety. It was associated with all functional neuroimaging modalities, but not the structural modalities, in the amygdala, thalamus, and insula with involvement of the anterior salience network. Anxiety symptoms are frequently present in psychiatric disorders, particularly in internalizing and thought disorders.^22^ The correlation between the MID component score and the MID activation is the inverse of the depressive mood and distress score. The differences in loading represent the variance in MID that separates depressive mood from anxiety rather than their comorbidity. This specificity enables the potential targeting of biological features for possible mechanistic intervention.

The stress pathology score (component 4) was associated with the SST with the strongest positive loading in the anterior cingulate cortex consistent with hyperactivation in this region associated with emotional reactivity and vigilance. The clinical items contributing to the scores were predominately related to posttraumatic stress disorder, suggesting a link to prior trauma. This association is bolstered by the pivotal role of the anterior cingulate cortex in emotional reactivity and posttraumatic stress disorder.^23^

The eating pathology score (component 5) was associated with the resting-state brain modes and particularly connectivity between the ventral default mode, basal ganglia, and temporal networks. Both functional and structural associations with the temporal lobe have been reported in bulimia nervosa, where these networks are thought to be associated with social behavior and emotional stimuli.^24^

Social fear and avoidance score (component 6) was only associated with resting-state brain modes, particularly with respect to connectivity in the dorsal default mode and left executive control networks, suggesting neural mechanisms underlying deficits in cognitive control during experiences of fear.^25^

For each psychopathology component individually, the variance explained by the neuroimaging variates was moderate, which limits the utility of these neuroimaging variates in a clinical setting. Our objective was to identify behavioral symptom groups informed by their underlying biology. The psychopathology features exhibited the highest covariance across clinical items and multimodal features simultaneously. These clinical and MRI features are orthogonal to each other, meaning that each subsequent component explains the residual variance not accounted for by the previous component. This approach arguably parses both clinical and biological heterogeneity. Consequently, our model offers a more precise understanding by directly identifying biomarkers associated with specific psychopathology components, free from the confounding effects of comorbidity.

### Limitations

There are limitations in translating these findings to clinical application. Our model was developed using a naturalistic sample, minimizing potential confounds from psychiatric treatment, such as medications, but likely missing out on psychiatric disorders with a lower prevalence (eg, schizophrenia) or older age at onset (eg, dementia). Further, all participants were of Western European origin, potentially limiting the generalizability of our findings to a more diverse population. Additionally, we have not mapped individual differences in the neuroimaging modalities to average brain functioning such as those used in normative modeling.^26^ Furthermore, norms need to be established in these models. SGCCA and similar data-driven approaches offer flexibility in determining statistical models, including sparsity methodology, feature selection, and covariance optimization functions. While beneficial, a major drawback is the lack of standardization—no 2 CCA models are alike. A consensus is necessary on which biomarkers to include. Valid arguments exist for incorporating other psychiatric biomarkers beyond neuroimaging, including circulating markers, genomic/epigenetic profiles, electrophysiology, and neurochemical markers. This consensus is also needed for the type of clinical data included because these models are highly sensitive to biases in clinical input. The number of items contributing to a symptom can disproportionately affect its weight in the overall model. We coded the DAWBA skip rules using zeros, assuming that omissions due to skip rules were rare in our samples.^8^ This approach potentially imposes a covariance structure on the clinical input; however, bias in such models is currently inevitable. If we had only used entry items, information about symptom severity and frequency would have been lost. Symptom questionnaires without skip rules are potentially biased toward a specific diagnosis. Therefore, while we do not claim that our choice of statistical parameters or input data is optimal, we provide proof-of-principle that a reliable model can be produced, emphasizing the need for methodological consensus before these models are ready for clinical application. Therefore, our methodology in enriched datasets and patient populations may have more concrete implications for mental illness treatment.

## Conclusions

In conclusion, jointly linking psychiatric symptoms to multimodal brain features lays the groundwork for a dimensional approach to psychiatry optimized for brain biomarkers. We present proof of principle for a framework that points to quantifiable neurobiological measures enabling precise targeting of biological features for mechanistic intervention. Our results demonstrate the feasibility of SGCCA methodology to produce stable brain-linked psychopathology features but also highlight the need to have consensus among clinical and biological parameters for clinical application. Our framework for neurobiology-enhanced dimensions of psychopathology may enable quantitative assessment of comorbidity necessary for precision medicine and demonstrates their potential to bridge the gap between psychiatric neuroscience and clinical treatment of mental disorders.

## Notes

Supplement 1. eMethods eAppendix. STROBE checklist eTable 1. IMAGEN exclusion criteria eTable 2. STRATIFY/ESTRA exclusion criteria eTable 3. Sample counts for each data view eFigure 1. Ward’s hierarchical clustering of the 14 resting state network edges and correlation matrix of the dual regression synthetic time series eFigure 2. The parcels derived from Ward’s hierarchical clustering eFigure 3. Sparse generalized canonical correlation analysis (SGCCA) model optimization, variable selection, and optimized model assessment eFigure 4. Longitudinal association between psychopathology scores and neuroimaging scores in training and test data

Supplement 2. environMENTAL Consortium members

Supplement 3. Data sharing statement
