# Supplementary material for: Framework for Brain-Derived Dimensions of Psychopathology
Source: JAMA Psychiatry. 2025 Jun 18;82(8):778–89. doi: 10.1001/jamapsychiatry.2025.1246 (PMC12177734; doi:10.1001/jamapsychiatry.2025.1246)
Supplement: Supplement 1. — eMethods eAppendix. STROBE checklist eTable 1. IMAGEN exclusion criteria eTable 2. STRATIFY/ESTRA exclusion criteria eTable 3. Sample counts for each data view eFigure 1. Ward’s hierarchical clustering of the 14 resting state network edges and correlation matrix of the dual regression synthetic time series eFigure 2. The parcels derived from Ward’s hierarchical clustering eFigure 3. Sparse generalized canonical correlation analysis (SGCCA) model optimization, variable selection, and optimized model assessment eFigure 4. Longitudinal association between psychopathology scores and neuroimaging scores in training and test data [file jamapsychiatry-e251246-s001.pdf]

Supplemental Online Content

Lett TA, Vaidya N, Jia T, et al; IMAGEN Consortium; environMENTAL Consortium. Framework for brain-derived dimensions of psychopathology. *JAMA Psychiatry*. Published online June 18, 2025. doi:10.1001/jamapsychiatry.2025.1246

eMethods
eAppendix. STROBE checklist
eTable 1. IMAGEN exclusion criteria
eTable 2. STRATIFY/ESTRA exclusion criteria
eTable 3. Sample counts for each data view
eFigure 1. Ward's hierarchical clustering of the 14 resting state network edges and correlation matrix of the dual regression synthetic time series
eFigure 2. The parcels derived from Ward's hierarchical clustering
eFigure 3. Sparse generalized canonical correlation analysis (SGCCA) model optimization, variable selection, and optimized model assessment
eFigure 4. Longitudinal association between psychopathology scores and neuroimaging scores in training and test data

This supplemental material has been provided by the authors to give readers additional information about their work.

© 2025 Lett TA et al. *JAMA Psychiatry*.

eMethods

Literature search

Meta-analyses have demonstrated heterogeneous neuroimaging associations with psychiatric disorders. The absence of concrete relationships between clinical criteria and brain biomarkers had been a barrier to the development of novel treatments. We searched PubMed from Jan 1, 2000 to May 30, 2024, with the terms ((“cross-disorder”) OR (“psychopathology”) OR (“co-morbidity”) OR (“comorbidity”) OR (“nosology”)) AND ((“neuroimaging”) OR (“fMRI”) OR (“functional MRI”) OR ((“surface area”) OR (“cortical thickness”) OR (“fractional anisotropy”) OR (“white matter integrity”) OR (“resting state”) OR (“connectivity”)) AND ((“meta-analysis”) OR (“population-based”) OR (“RDoc”) OR (“HiTOP”) OR (“canonical correlation analysis”)). No language restrictions were applied to this search. We found that most studies are cross-sectional, limited to a single psychiatric diagnosis, or rely on questionnaires with limited overlaps. There were no studies that comprehensively covered the wide spectrum of clinical symptoms derived from ICD-10, ICD-11, or DSM-V to brain structure, function, and connectivity.

Cohort assessments

The cohorts include predominantly White, Europeans (self-described and validated using principal component analysis of population stratification informative genetic variants [1] from eight clinical research hospitals in: England, France, Germany, and Ireland. IMAGEN includes extensive clinical, biological, and assessments that have been previously described [2]. Each research site received approval from the relevant local research ethics committee in accordance with the Declaration of Helsinki. Written consent was obtained from each participant and a parent or guardian. The Development and Wellbeing Assessment (DAWBA)[3], Strengths and Difficulties Questionnaire (SDQ)[4], and Alcohol Use Disorders Identification Test (AUDIT) [5], as well as neuroimaging assessments performed in STRATIFY and ESTRA were nearly identical to IMAGEN’s assessment. In addition to healthy controls (n=26), STRATIFY/ESTRA also contains participants meeting diagnostic criteria for patients diagnosed with: attention deficit hyperactivity disorder (ADHD; n=1), alcohol use disorder (AUD; n=64), major depressive disorder (MDD; n=78), and psychosis (n=3) (i.e., STRATIFY sample), and anorexia nervosa (AN; n=22), bulimia nervosa (BN; n=19) (i.e., ESTRA sample). Due to the small sample size of the ADHD and psychosis groups with complete data, they were excluded from the present study.

Clinical psychiatric symptoms were assessed using the DAWBA, SDQ, and AUDIT. DAWBA screening questions have previously been used to define subthreshold clinical symptoms in neuroimaging studies of psychopathology[3,6]. The SDQ was also used in the present investigation, as this questionnaire contributes to the assignment of diagnostic status in the DAWBA[4]. The AUDIT questionnaire was used to screen for potential harmful drinking and identify mild dependence[5]. The DAWBA, SDQ, and AUDIT focus primarily on current psychopathology, primarily within the last four weeks. We were interested in the symptoms rather than diagnostic criteria; therefore, we considered all items in these questionnaires rather than only the entry items. We posit that this strategy allows us to describe precise interrelationships among different categories of questions. A consequence of using all items in structural interviews means that we treat questions that have not been asked as having no symptoms. In total, there were 335 items among all questionnaires after two items from the DAWBA were dropped because they had zero variance across the training sample.

Ethics approval

Written and informed consent was obtained from all participants by the IMAGEN consortium and the study was approved by: The institutional ethics committee of King's College London (PNM/10/11-126), University of Nottingham (D/11/2007), Trinity College Dublin (SPREC092007-01), Technische Universitat Dresden (EK 235092007), Commissariat a l'Energie Atomique et aux Energies Alternatives, INSERM (2007-A00778-45), University Medical Center at the University of Hamburg (M-191/07) and in Germany at medical ethics committee of the University of Heidelberg (2007-024N-MA) in accordance with the Declaration of Helsinki. Written

© 2025 Lett TA et al. *JAMA Psychiatry.*

and informed consent for STRATIFY was obtained from all participants and the study was approved by Charité – Universitätsmedizin Berlin (EA1/030/18) in Germany and the NHS Health Research Authority (IRAS ID – 218030) for King's College London (17/LO/0552) and University of Southampton (RHM MED1439).

Image acquisition

MRI scans were acquired from 3-Tesla scanners from different manufacturers (Siemens, Munich, Germany; Philips, Best, The Netherlands; General Electrics, Chalfont St Giles, UK; Bruker, Ettlingen, Germany) at eight different sites (King's College, London; Sir Peter Mansfield Imaging Centre of the University of Nottingham; Trinity College Institute of Neuroscience, Dublin; the Centre de Neuroimagerie de Recherche, Paris; Charité Universitätsmedizcine Berlin; Universitätsklinikum Hamburg-Eppendorf; Zentralinstitut für seelische Gesundheit, Mannheim; and the Universitätsklinikum Carl Gustav Carus, Dresden). Acquisition protocols for sMRI, DWI, resting state fMRI (rs-fMRI), as well as monetary incentive delay (MID), emotional faces task (EFT), and stop signal task (SST) as described in previous publications [1,2,7,8]. In brief, high-resolution anatomical MRIs were acquired, including a three-dimensional (T1-weighted; sagittal slice plane; repetition time (TR) 2.3s; echo time (TE) 2.93ms; flip angle 9°; 256×256×160 matrix; isotropic voxel size 1.1mm) magnetization prepared gradient echo sequence (MPRAGE) based on the ADNI protocol. DWI image acquisition was identical across sites using an echo planar imaging (b=0 and 32 directions with b-value 1,300 $s*mm^{-2}$; axial slice plane; echo time = 104ms; 128×128×60 matrix; voxel size 2.4×2.4×2.4mm). The fMRI standardized acquisition parameters were performed using a single-shot T2*-weighted gradient-echo echo planar imaging (GE-EPI) sequence with 3.4 x 3.4 mm in-plane resolution, 164 volumes, 40 slices with a thickness of 2.4 mm with a gap of 3.4 mm, TR of 2200 ms, TE of 30 ms, a flip angle of 75°, and a Field of View of 218 x 218 mm. Volumes were acquired in sequential ascending slice order. For rs-fMRI, participants were instructed to close eyes and to not focus on a specific thought (5-6 min duration). All imaging data were visually screened for corrupted data or acquisition artifacts. For the STRATIFY sample, the identical MRI acquisitions were performed at three of the eight sights (King's College, London; Charité Universitätsmedizcine Berlin; and University of Southampton, Southampton)

Processing of structural images

Cortical reconstruction was performed on all T1-weighted images using the Freesurfer image analysis suite (http://surfer.nmr.mgh.harvard.edu/). The technical details of these procedures are described in prior publications [9–13]. In brief, this process includes motion correction and averaging of multiple volumetric T1 weighted images, removal of non-brain tissue, automated Talairach transformation, segmentation of the subcortical white matter and deep gray matter volumetric structures, intensity normalization, tessellation of the gray matter white matter boundary, automated topology correction, and surface deformation. A number of deformable procedures were performed including surface inflation, registration to a spherical atlas which is based on individual cortical folding patterns to match cortical geometry across subjects, and creation of a variety of surface-based data including maps of curvature and sulcal depth. Both intensity and continuity information from the entire three-dimensional MR volume in segmentation and deformation procedures to produce representations of cortical thickness, calculated as the closest distance from the gray/white boundary to the gray/CSF boundary at each vertex on the tessellated surface [9].

Processing of Diffusion Weighted Images

Diffusion data preprocessing was performed using tools provided by the MRtrix3 software package http://mrtrix.org (Tournier et al., 2019), including, for some preprocessing step, scripts interfacing with the external package FSL FMRIB Software Library (FSL) https://fsl.fMRIb.ox.ac.uk/fsl/fslwiki/. Diffusion images were denoised (Veraart et al., 2016), corrected for Gibbs ringing artifacts (Kellner et al., 2016), and corrected for eddy current-induced distortions and subject movements (Andersson and Sotiropoulos, 2016) using outlier replacement (Andersson et al., 2016) and a linear second level model. Then, a B1 field inhomogeneity correction was performed using the N4 algorithm as provided in ANTs (Turtison et al, 2010). The diffusion tensor model was fitted using a weighted least-squares estimator (Veraart et al., 2016) before extracting derivative diffusion metrics (FA, MD, AD and RD). Following methodological recommendations on diffusion tensor image registration [14] and on tract-based spatial statistic [15], We used a tensor-based registration with DTI-TK http://dti-tk.sourceforge.net/pmwiki/pmwiki.php [16] to align all individuals into a common space. The multiple time-point images were processed within an unbiased longitudinal framework using tensor-based registration [17]. The first step create a within-subject template based on the

© 2025 Lett TA et al. *JAMA Psychiatry.*

multiple time point images per subject. Then a second step created a group-wise atlas based on the within-subject templates. At the end of the registration procedure, each participant's diffusion data were normalized to the MNI standard space using the IIT human brain tensor template [18]. Voxel-wise statistical inference of the diffusion tensor metrics was carried out using the TBSS procedure [19]. From all previously obtained images, the mean FA image was created and thinned to create a mean FA skeleton, which represents the centers of all tracts common to the group. This skeleton was then thresholded to FA > 0.2 to keep only the main tracts. Each participant's aligned fractional anisotropy images were then projected onto this common skeleton to minimize any residual misalignment of tracts.

Task-based fMRI

To assess fMRI blood-oxygen-level-dependent (BOLD) activation in responses to reward anticipation and reward outcome [20], the participants performed the monetary incentive delay (MID) fMRI task. In 66 ten-second trials, participants were presented with one of three cue shapes (cue, 250 ms) denoting whether a target (white square) would subsequently appear on the left or right side of the screen and whether 0, 2, or 10 points could be won in that trial. After a variable delay (4,000-4,500 ms) of fixation on a white crosshair, participants were instructed to respond with left/right button-press as soon as the target appeared. Feedback on whether and how many points were won during the trial was presented for 1450 ms after the response. Our outcome contrast for this task was large-win versus no-win.

For fMRI BOLD response to emotional stimuli, we used the emotional face task (EFT) [21]. Participants watched 18-second blocks of either a face movie (depicting anger or neutrality) or a control stimulus. Each face movie showed black and white video clips (200-500ms) of male or female faces. Five blocks each of angry and neutral expressions were interleaved with nine blocks of the control stimulus. Each block contained eight trials of 6 face identities (3 female). The same identities were used for the angry and neutral blocks. The control stimuli were black and white concentric circles expanding and contracting at various speeds that roughly matched the contrast and motion characteristics of the face clips. As in previous IMAGEN studies, our primary contrast was viewing angry faces (vs control) [8].

Last, we assess fMRI BOLD activation during response inhibition using the stop-signal task [22]. The task was composed of Go trials and Stop trials that activate top-down attentional control, particularly in the inferior frontal cortex. During Go trials (83%; 480 trials) participants were presented with arrows pointing either to the left or to the right, and subjects were instructed to make a button response with their left or right index finger corresponding to the direction of the arrow. In the unpredictable Stop trials (17%; 80 trials), the arrows pointing left or right were followed (on average 300 ms later) by arrows pointing upward and subjects were instructed to inhibit their motor responses during these trials. A tracking algorithm changes the time interval between Go signal and Stop signal onsets according to each subject's performance on previous trials (average percentage of inhibition over previous Stop trials, recalculated after each Stop trial), resulting in 50% successful and 50% unsuccessful inhibition trials. The inter-trial interval was 1800 ms. The tracking algorithm of the task ensured that subjects were successful on 50% of Stop trials and worked at the edge of their own inhibitory capacity. We assessed the stop success contrast in accordance with previous IMAGEN publications [8].

Task-based fMRI processing

Task-based functional MRI data were analyzed with SPM8 (Statistical Parametric Mapping; http://www.fil.ion.ucl.ac.uk/spm). Spatial preprocessing included: slice time correction to adjust for time differences due to multi-slice imaging acquisition, realignment to the first volume in line, non-linearly warping to the MNI space (based on a custom EPI template (53x63x46 voxels) created out of an average of the mean images of 400 adolescents), resampling at a resolution of 3x3x3 mm^3 and smoothing with an isotropic Gaussian kernel of 5 mm full-width at half-maximum.

Resting-state fMRI processing

FEAT (fMRI Expert Analysis Tool) Version 6.00 in FSL was used to perform data preprocessing on the functional data, including motion correction [23], slice time correction, BET, spatial smoothing using a Gaussian kernel of 4mm FWHM, and grand mean intensity normalization. Independent Component Analysis (ICA)-based automatic removal of motion-related and physiological noise artifacts was used to further clean the data (ICA-AROMA; [24]). High-pass temporal filtering (> 0.008 Hz) was applied to remove slow drifts. The middle EPI volume was co-registered to the individual brain-extracted T1 image using boundary-based registration[25]. Non-linear normalization of the T1 image to the 3mm MNI standard space template (Montreal Neurological Institute, Quebec, Canada) was done using

© 2025 Lett TA et al. *JAMA Psychiatry.*

ANTs [26]. The preprocessed data were normalized to 3mm MNI standard space, applying the registration matrices and warp images from the two previous registration steps. Within-subject time-series were corrected for using Nilearn (http://nilearn.github.io) with white matter and cerebrospinal fluid mean activation as covariates.

Creation of resting-state network matrices

Resting-state networks were created using FSL's melodic group independent component analysis (GICA) and network modeling using stage-1 dual regression and the FSLNets toolbox (http://fsl.fMRIb.ox.ac.uk/fsl/fslwiki/FSLNets). Initially, GICA was performed by temporal concatenation of the time-series data from 400 adolescents from IMAGEN to generate 25 independent components. Of those 25 components, 14 were deemed to represent known resting state networks (Supplementary Figure 1). Of note, as little as five dimensionalities are needed to produce similar canonical correlation analysis results as high dimension networks [27]. The networks were identified using a functional atlas [28] and included the: Anterior Salience Network, Auditory Network, Basal Ganglia Network, Dorsal Default Mode Network, Higher Visual Network, Language Network, Left Executive Control Network, Sensorimotor Network, Posterior Salience Network, Precuneus Network, Primary Visual Network, Right Executive Control Network, Ventral Default Mode Network, Visuospatial Network (Supplementary Figure 2). From these 14 networks, FSL's dual_regression script extracted time-series for each component which was used in FSLNets to calculate a correlation matrix for each subject. In total, there were 91 pairwise correlations (upper-right triangle) that underwent Fischer's Z-transformation (inverse hyperbolic tangent) to approximately variance-stabilize the correlation coefficients. We did not employ L2 regularization (ridge) because our multiple sparse canonical correlation analysis (see below) already imposes L1 regularization.

Data driven clustering of task-based fMRI and tract-based spatial statistics

Given that there is no standard atlas for our fMRI tasks, and the Johns Hopkins University diffusion-based white-matter atlas [29,30] does not provide full coverage of our average study tract-based spatial statistics (TBSS) skeleton, we employed hierarchical agglomerative clustering [31] using Ward's algorithm for variance minimization [32]. We selected this model of hierarchical cluster over other alternatives (e.g., k-means), because for task-based fMRI data Ward's algorithm performed better than many other geometric clustering methods across a variety of configurations [33]. Agglomerative clustering was performed using scikit-learn (https://scikit-learn.org/). First, to discard low-variance noise components, we initially performed a principal component analysis to extract the top 100 components across all subjects and create a new projected 4D image. Then, using a group data mask, we calculated the adjacency matrix for each voxel in all directions (26 neighbors) and converted it to a sparse matrix in coordinate format. Next, using the de-noised 4D image and the sparse adjacency matrix we applied agglomerative clustering. For the fMRI, we choose 300 clusters within the recommended range of 200-500 (Supplementary Figure 3)[33]. Since we are applying sparsity to the mean values of the clusters, we included whole brain clustering as an additional check to our space canonical correlation model. That is, white matter and cerebrospinal fluid area will be removed from our model. For the TBSS skeleton, we chose 100 clusters since fewer anatomical clusters are likely sufficient for diffusion-based signals (Figure S3). Nevertheless, optimizing the number of parcels is still an open question [33], especially with respect to TBSS derived metrics.

Sparse Generalized Canonical Correlation Analysis (SGCCA) data views

There are a total of eight data views with similar dimensionality (ranging from 91 to 358 variables) including: the clinical scores (335 variables), the EFT task (300 variables), MID task (300 variables), SST task (300 variables), surface area (358 variables), cortical thickness (358 variables), white matter FA (100 variables), and the resting state networks (91 variables). The clinical data were transformed by a negative log transformation ($f(x) = sign(x) * log10(|x| + 1)$ because the clinical items were left skewed. Since the data views have an unequal number of variables, we weighted each view by dividing by the question of the number of variables. The effect of sex and site was regressed from all data views using Theil-Sen regression [34,35]. We used the Theil-Sen estimator because it is a non-parametric approach that is robust against potential bias from outliers.

Estimation of optimal SGCCA hyperparameters

We used a Python wrapper code (https://github.com/trislett/sparsemodels) that imports the core RGCCA implementation of the R Penalized Multivariate Analysis (RGCCA) package [36,37], and expands on its functionality.

© 2025 Lett TA et al. *JAMA Psychiatry.*

For model optimization, we applied a factorial functional scheme ($g(x) = x^2$) that maximizes the covariances [38]. One advantage of the RGCCA package is that it yields orthogonal block (view) components and orthogonal weight vectors (coefficients) for multiview data analyses.

The IMAGEN FU3 sample was divided into training data (70%) and test data (30%) with equal sampling across the eight sites (Figure 1). Within the training data (n = 559), we optimized the L_1 sparsity parameter using a permutation scheme for the first component [39]. We tested ten steps of lambda values ranging from 0.1 to 1.0 in 0.1 steps. For each step, we compared the objective function value of the true model to the rank-ordered permuted models to calculate significance, and z-scores for each step were calculated by dividing true values by the standard deviation of the permuted estimates. The best sparsity hyperparameter was selected according to the maximum Z-score. After estimating the optimal sparsity, we then determined the approximate optimal number of components by fitting the SGCCA model with initially 50 components. There is no standard, objective method for selecting the number of components for any matrix factorization method. Here, we used a similar approach to a principal component analysis, in which we defined the approximate optimal number of components at the point in which the "elbow" of the cumulative average variance is explained. After we had estimated the optimal sparsity and number of components, we then applied stability selection. Stability selection is generally applied to sparse CCA since the L_1 penalties applied to intercorrelated variables can be arbitrary in terms of selection and dependent on prior structure. We choose arguably the most established procedure [40] that has previously been applied in the IMAGEN sample [7]. In brief, a subsampling strategy is performed in which half of the subjects are randomly selected, the SGCCA model is fit and the variables with non-zero coefficients are saved. The process is repeated 10000 times, and only variables that are selected in 90% of subsampled models are considered stable. Last, a final SGCCA model is fitted to the training data stability selected variables, the optimal number of components, and without any L1 sparsity.

Significance of the SGGCA model

After model optimization, we employed a permutation scheme to compare the canonical correlates of the real data SGCCA model and rank them against 10,000 permutations of permuted (null) SGCCA models (Figure 1). We calculated the average inner variance explained (AVE_{inner}), represented by the mean of canonical correlations. The real data canonical variates were then ranked against the null models to determine their permuted p-values. To rule out possible overfitting of the model, we performed an additional assessment of significance using independent test data. We utilized the SGCCA model derived from the real training data and applied it to the test data. This involved taking the dot product of the training model coefficients and the test data views to calculate their canonical correlates. Subsequently, we compared the predicted canonical correlates in the test data to the predicted canonical correlates obtained from the 10000 permuted SGCCA models, rank ordering them for significance.

We considered a component to be of interest if the AVE_{inner} was significant in both the training data and the test data. We did not assess the significance of the model components alone, as the average inner canonical correlations would encompass correlations among all data view scores equally. Our primary focus was on the association between the clinical components and the other seven neuroimaging data views. We employed a combined regression and canonical correlation analysis SGCCA approach, which is a widely used and well-established method in multiview canonical correlation analyzes[7,37]. The combined approaches allowed us to specifically examine the association between the clinical components and the neuroimaging data views of interest. We conducted SGCCA-regression analyses to investigate the relationships between the latent clinical component (response variable) and the latent neuroimaging components (predictor variables), separately in the training and test IMAGEN data. The identical method was applied at the 14 and 19 time points of the IMAGEN training and test subjects as well as in STRATIFY/ESTRA. To determine the significance of the model and the coefficients, we employed 10,000 bootstraps. Components of interest were deemed significant if the regression model showed significance in both the training and test data.

We assessed the model loadings in the training data, which represent the correlation between the view components and their respective variables. These loadings indicate the amount of variance explained by each variable. The significance and confidence intervals of the loadings were evaluated using 10,000 bootstraps and Benjamin-Hochberg false discovery rate correction (FDR)[41].

© 2025 Lett TA et al. *JAMA Psychiatry.*

eAppendix
STROBE checklist

	Item No	Recommendation	
**Title and abstract**	1	(a) Indicate the study's design with a commonly used term in the title or the abstract	✓
		(b) Provide in the abstract an informative and balanced summary of what was done and what was found	✓
**Introduction**			
Background/rationale	2	Explain the scientific background and rationale for the investigation being reported	✓
Objectives	3	State specific objectives, including any prespecified hypotheses	✓
**Methods**			
Study design	4	Present key elements of study design early in the paper	✓
Setting	5	Describe the setting, locations, and relevant dates, including periods of recruitment, exposure, follow-up, and data collection	✓
Participants	6	(a) Cohort study—Give the eligibility criteria, and the sources and methods of selection of participants. Describe methods of follow-up<br>Case-control study—Give the eligibility criteria, and the sources and methods of case ascertainment and control selection. Give the rationale for the choice of cases and controls<br>Cross-sectional study—Give the eligibility criteria, and the sources and methods of selection of participants	✓
		(b) Cohort study—For matched studies, give matching criteria and number of exposed and unexposed<br>Case-control study—For matched studies, give matching criteria and the number of controls per case	N/A
Variables	7	Clearly define all outcomes, exposures, predictors, potential confounders, and effect modifiers. Give diagnostic criteria, if applicable	✓
Data sources/ measurement	8*	For each variable of interest, give sources of data and details of methods of assessment (measurement). Describe comparability of assessment methods if there is more than one group	✓
Bias	9	Describe any efforts to address potential sources of bias	✓
Study size	10	Explain how the study size was arrived at	✓
Quantitative variables	11	Explain how quantitative variables were handled in the analyses. If applicable, describe which groupings were chosen and why	✓
Statistical methods	12	(a) Describe all statistical methods, including those used to control for confounding	✓
		(b) Describe any methods used to examine subgroups and interactions	✓

© 2025 Lett TA et al. *JAMA Psychiatry.*

		(c) Explain how missing data were addressed	✓
		(d) Cohort study—If applicable, explain how loss to follow-up was addressed<br>Case-control study—If applicable, explain how matching of cases and controls was addressed<br>Cross-sectional study—If applicable, describe analytical methods taking account of sampling strategy	✓
		(e) Describe any sensitivity analyses	✓
**Results**			
Participants	13*	(a) Report numbers of individuals at each stage of study—eg numbers potentially eligible, examined for eligibility, confirmed eligible, included in the study, completing follow-up, and analysed	✓
		(b) Give reasons for non-participation at each stage	✓
		(c) Consider use of a flow diagram	N/A
Descriptive data	14*	(a) Give characteristics of study participants (eg demographic, clinical, social) and information on exposures and potential confounders	✓
		(b) Indicate number of participants with missing data for each variable of interest	✓
		(c) Cohort study—Summarise follow-up time (eg, average and total amount)	✓
Outcome data	15*	*Cohort study—Report numbers of outcome events or summary measures over time*	✓
		Case-control study—Report numbers in each exposure category, or summary measures of exposure	N/A
		Cross-sectional study—Report numbers of outcome events or summary measures	✓
Main results	16	(a) Give unadjusted estimates and, if applicable, confounder-adjusted estimates and their precision (eg, 95% confidence interval). Make clear which confounders were adjusted for and why they were included	✓
		(b) Report category boundaries when continuous variables were categorized	✓
		(c) If relevant, consider translating estimates of relative risk into absolute risk for a meaningful time period	✓
Other analyses	17	Report other analyses done—eg analyses of subgroups and interactions, and sensitivity analyses	✓
**Discussion**			
Key results	18	Summarise key results with reference to study objectives	✓

© 2025 Lett TA et al. *JAMA Psychiatry.*

Limitations	19	Discuss limitations of the study, taking into account sources of potential bias or imprecision. Discuss both direction and magnitude of any potential bias	✓
Interpretation	20	Give a cautious overall interpretation of results considering objectives, limitations, multiplicity of analyses, results from similar studies, and other relevant evidence	✓
Generalisability	21	Discuss the generalisability (external validity) of the study results	✓
**Other information**			
Funding	22	Give the source of funding and the role of the funders for the present study and, if applicable, for the original study on which the present article is based	✓

eTable 1. IMAGEN Exclusion Criteria

Category	Item	Action
Demographics	Child in target age (14 years)	Inclusion
	Self-reported Western European ancestry	Inclusion
Pregnancy and birth	Use of alcohol by the mother during pregnancy (>210 ml alcohol/week)	Exclusion
	Diabetes of the mother during pregnancy (onset before pregnancy, treated by insulin)	Exclusion
	Premature birth (< 35 weeks) and/or detached placenta	Exclusion
	Hyperbilirubinemia requiring transfusion	Exclusion
Child’s medical history	Type 1 diabetes	Exclusion
	Systemic rheumatologic disorders	Exclusion
	Malignant tumors requiring chemotherapy	Exclusion
	Congenital heart defects or heart surgery	Exclusion
	Aneurysm	Exclusion
Neurological conditions	Epilepsy	Exclusion
	Bacterial Infection of CNS	Exclusion
	Brain tumor	Exclusion
	Head trauma with loss of consciousness >30 minutes	Exclusion
	Muscular dystrophy, myotonic dystrophy	Exclusion
Developmental conditions	Nutritional and metabolic diseases	Exclusion
	Major neuro-developmental disorders (e.g. autism spectrum disorders)	Exclusion
	Hearing deficit	Exclusion
	Vision problems	Exclusion
Mental health and abilities	Treatment for schizophrenia, bipolar disorder	Exclusion
	IQ < 70	Exclusion

© 2025 Lett TA et al. *JAMA Psychiatry.*

MR contraindications	Metal implants	Exclusion
	Electronic implants (e.g. pacemakers)	Exclusion
	Severe claustrophobia	Exclusion

CNS, central nervous system, IQ, Intelligence quotient

eTable 2. STRATIFY/ESTRA Exclusion Criteria

**Category**	**Item**	**Action**
Demographics	Young adults in target age (18 to 30)	Inclusion
	Self-reported Western European ancestry	Inclusion
Medical history	Type 1 or Type 2 diabetes	Exclusion
	Heavily medicated for serious illness (other than diagnosis investigation)	Exclusion
	Participants who are pregnant or any possibility that they may be pregnant	Exclusion
	Restricted mobility, including inability to lie flat for 1.5 hours	Exclusion
Neurological conditions	Epilepsy	Exclusion
	Bacterial Infection of CNS	Exclusion
	Brain tumor	Exclusion
	Head trauma with loss of consciousness >30 minutes	Exclusion
	Muscular dystrophy, myotonic dystrophy	Exclusion
Developmental conditions	Nutritional and metabolic diseases	Exclusion
	Hearing deficit (requiring hearing aid)	Exclusion
	Vision problems (visual deficit not correctable)	Exclusion
MR contraindications	Metal implants	Exclusion
	Electronic implants (e.g. pacemakers)	Exclusion
	Severe claustrophobia	Exclusion
Diagnosis Specific Criteria		
Category	Item	Action
Healthy Controls	PHQ-9 total score < 5	Inclusion
	AUDIT total score < 5	Inclusion
	No current/past mental health issues.	Inclusion
	No regular medication for serious physical health issues.	Inclusion
	No learning difficulties.	Inclusion
	No self-reported regular recreational drug use.	Inclusion
	No 1st or 2nd order family members with mental health issues.	Inclusion
Major Depressive Disorder	Current and acute, moderate - severe depression (PHQ-9 >= 15)	Inclusion
Alcohol Use Disorder	Moderate - severe alcohol abuse (AUDIT total score >= 15)	Inclusion
Psychosis	Schizophrenia, schizotypal and delusional disorders (F20-F29 ICD-10 diagnosis)	Inclusion

© 2025 Lett TA et al. *JAMA Psychiatry.*

	Minimum one-episode schizophreniform illness/ schizophrenia or chronic schizophrenia.	Inclusion
Anorexia nervosa	Current diagnosis by EDDS - DSM-5 Version	Inclusion
	BMI < 18.5 (based on self-report of current height and weight).	Inclusion
Bulimia nervosa	Current diagnosis by EDDS - DSM-5 Version	Inclusion

AUDIT, Alcohol Use Disorders Identification Test; BMI, body mass index; CNS, central nervous system; EDDS, Eating Disorder Diagnostic Scale; IQ, Intelligence quotient; PHQ-9, Patient Health Questionnaire-9

eTable 3. Sample counts for each data view

Measure	IMAGEN (BL)	IMAGEN (FU2)	IMAGEN (FU3)	STRATIFY/ESTRA
DAWBA-SDQ / AUDIT	2096	1300	1253	485
Emotional Face Task (fMRI)	1943	1403	1162	511
Stop-Signal Task (fMRI)	1901	1396	1157	510
Monetary Incentive Delay Task (fMRI)	1562	1339	1086	347
Cortical Surfaces (CT and SA)	2012	1308	1151	514
White Matter FA	1414	968	942	431
Resting-state fMRI	382	1065	1168	421
Complete Data*	202	683	794	209

AUDIT, Alcohol Use Disorders Identification Test; CT, cortical thickness; DAWBA, Development and Well-Being Assessment; fMRI, functional magnetic resonance imaging, SA, cortical surface area; SDQ, Strength and Difficulties Questionnaire. *The number of subjects with complete data from the clinical data view and all neuroimaging modalities.

eFigure 1. Ward's hierarchical clustering of the 14 resting state network edges and correlation matrix of the dual regression synthetic time series.

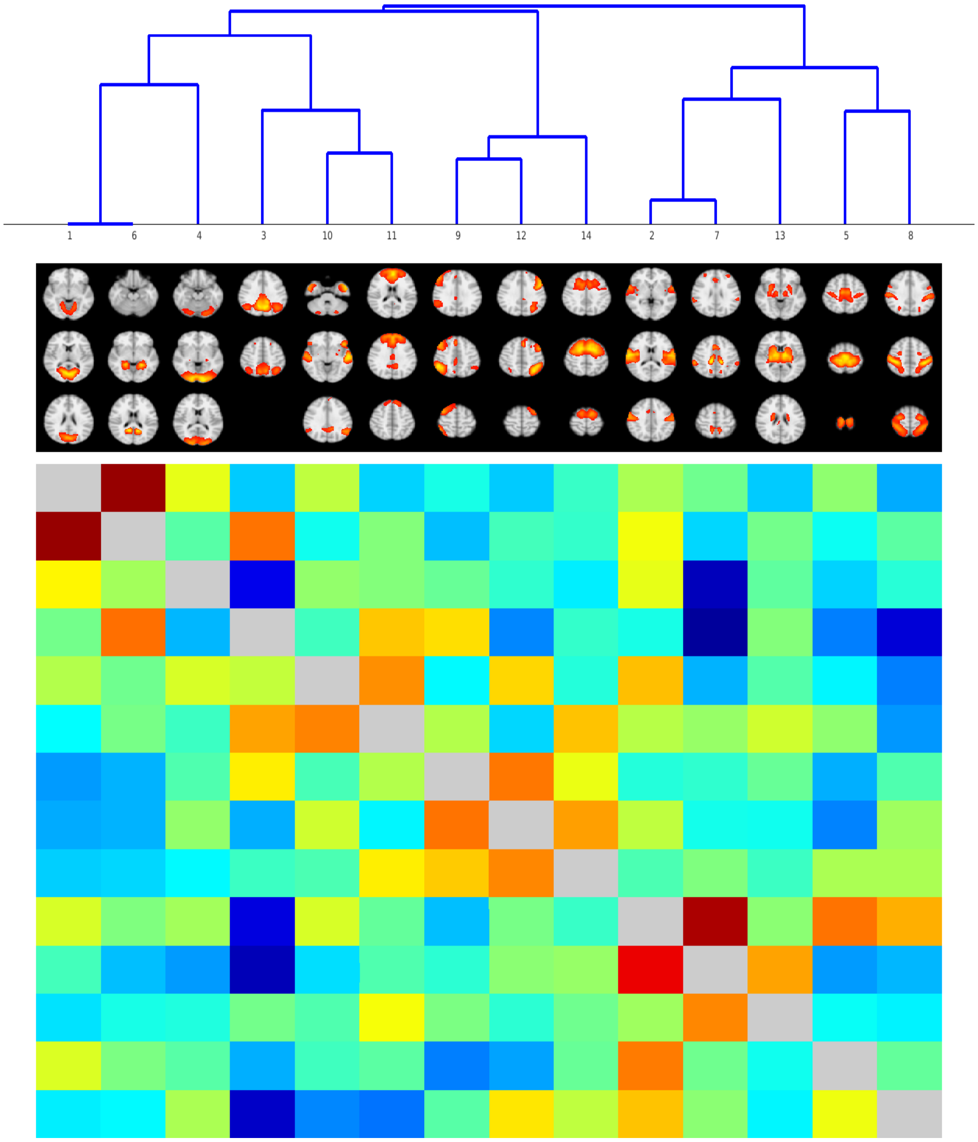

Identified resting state networks are numbered as the following: Anterior Salience Network (14), Auditory Network (2), Basal Ganglia Network (13), Dorsal Default Mode Network (11), Higher Visual Network (4), Language Network (10), Left Executive Control Network (12), Sensorimotor Network (5), Posterior Salience Network (7), Precuneus Network (3), Primary Visual Network (1), Right Executive Control Network (9), Ventral Default Mode Network (6), Visuospatial Network (8).

© 2025 Lett TA et al. *JAMA Psychiatry.*

eFigure 2. The parcels derived from Ward's hierarchical clustering.

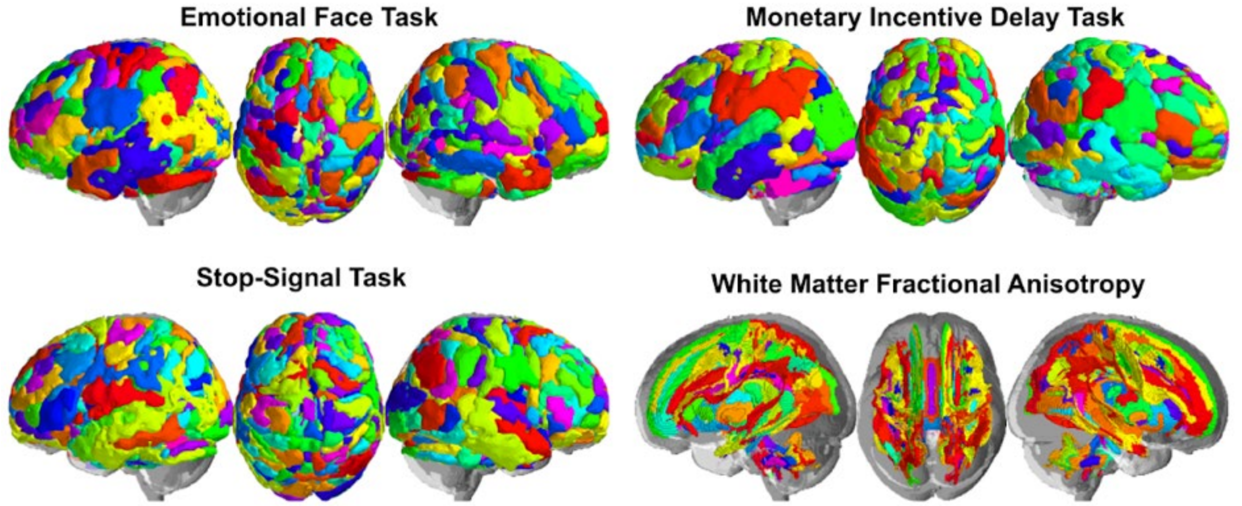

Ward's hierarchical clustering label for the emotional face fMRI task, monetary incentive delay fMRI, stop-signal fMRI task, and white matter fractional anisotropy TBSS skeleton. Each region of the atlases is assigned random colors.

© 2025 Lett TA et al. *JAMA Psychiatry.*

eFigure 3. Sparse generalized canonical correlation analysis (SGCCA) model optimization, variable selection, and optimized model assessment.

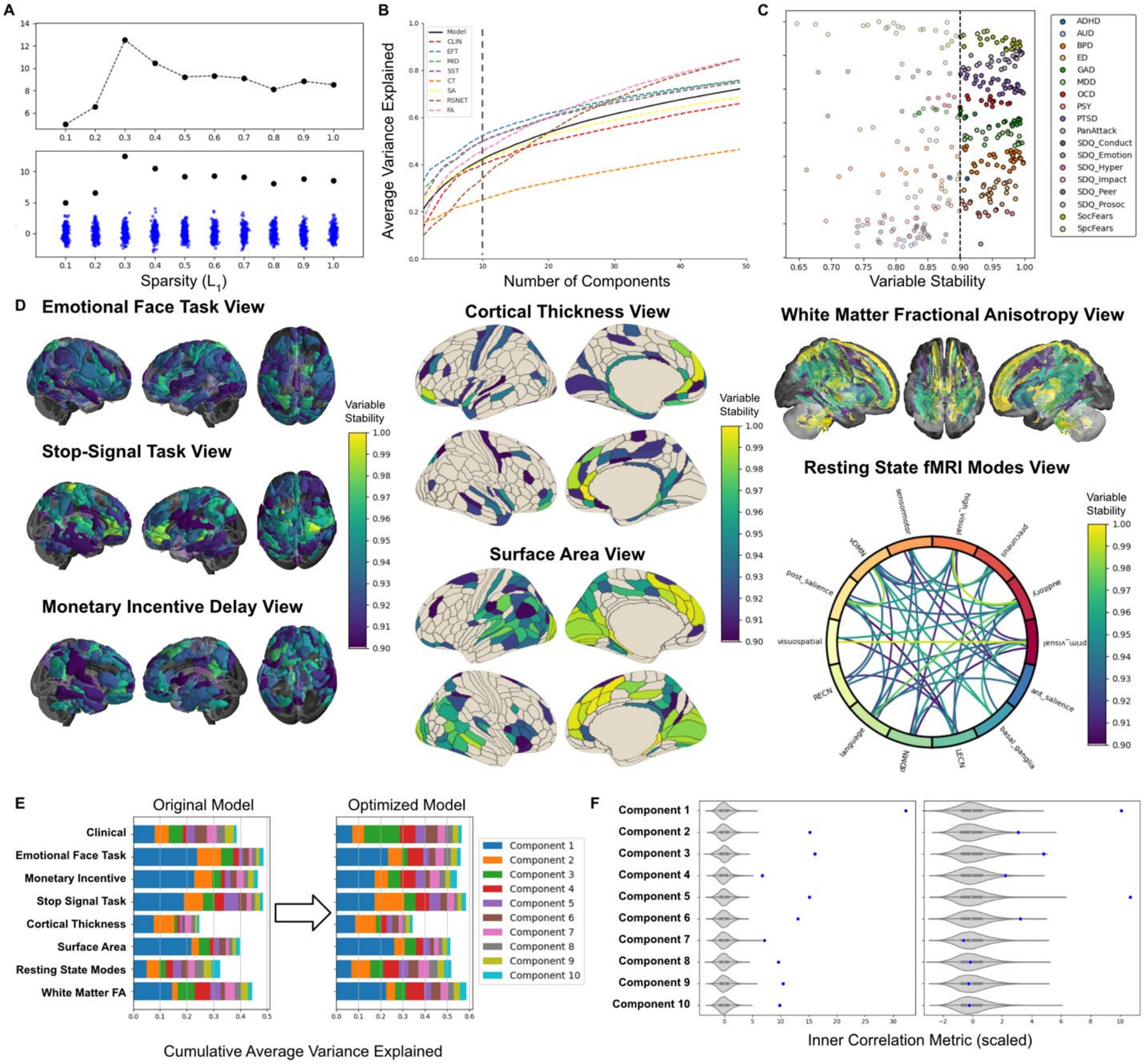

Line plots (A) depict the z-scores (top) and the scaled factorial objective function (bottom), which measures covariance among the data-views (black dots) and permuted data-views (blue dots) for different values of L_1 sparsity (λ_1) ranging from 0.1 to 1.0 in intervals of 0.1. The highest z-score (z = 12.6) was achieved at λ_1 = 0.3, indicating the optimal sparsity. Cumulative average variance explained (B; AVE) plotted against the number of components (x-axis) for the SGCCA model with 50 components. The solid black line represents the total model, and the dotted colored lines represent each data view. The selection of ten informative components (approximate elbow of the model curve) explains 40.4% of the cumulative variance among all data-views. Stability selection (C) by 10000 subsamples (random sampling 50% of the training data without replacement) retained the clinical items. A vertical dotted line separates variables kept in the clinical data view. Neuroimaging stability selection (D) by 10000 subsamples (random sampling 50% of the training data without replacement) retained the neuroimaging data-views that appeared in 90% of the subsampled SGCCA models. Colored regions indicate brain regions used in the optimized generalized canonical correlation analysis that appeared in 90% of the subsampled SGCCA models. Barplots of the AVE (E) for each data view aggregated along the x-axis for each component represented by different colors in the initial model (all variables, components = 10, and λ_1 = 0.3) and the final optimized model (stability selected

© 2025 Lett TA et al. *JAMA Psychiatry.*

variables, components = 10, and $\lambda_1 = 1.0$) in the training data. Violin plots showing the optimized permuted models for the inner average variance explained (AVE) in the training data and test sample (F). The AVE of the actual model are blue dots. Using the selected variables, our actual model and 10000 permuted (null) models were created. The components are considered significant if the actual model AVE is greater than 95% of the permuted models. All components in the training model were significant, whereas only the first six components were significant in the training data ($p_{permuted} < 0.05$).

© 2025 Lett TA et al. *JAMA Psychiatry.*

eFigure 4. Longitudinal association between psychopathology scores and neuroimaging scores in training and test data.

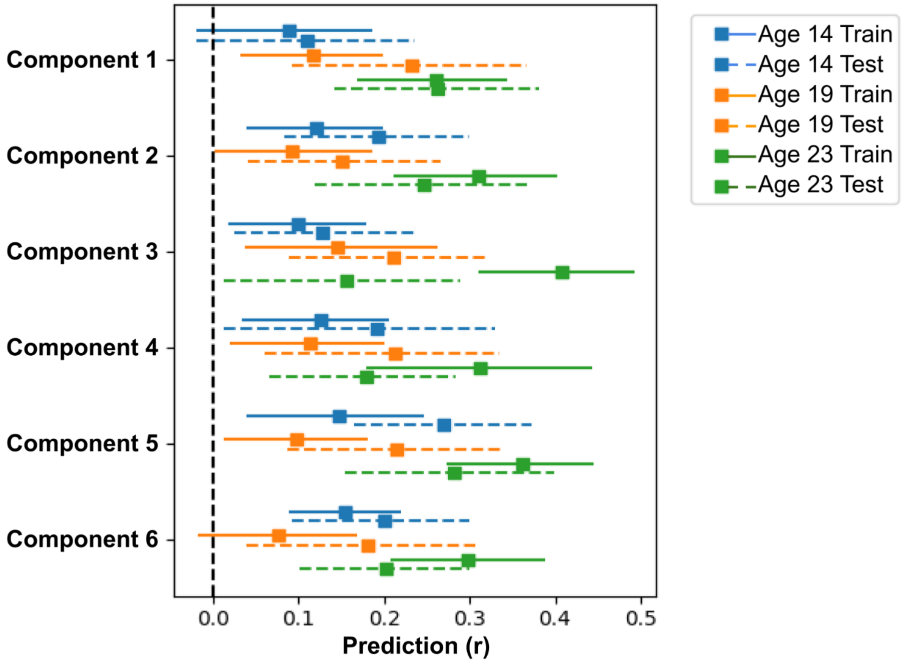

To evaluate the longitudinal relationship between clinical symptoms and neuroimaging features, SGCCA-regression was employed. The endogenous variables were the clinical view scores, and the neuroimaging view scores were the exogenous variables corresponding to each component. The figure includes bootstrapped 95% confidence intervals represented by blue, yellow, and red lines at ages 14, 19, and 23. Bootstrapped confidence intervals that do not cross the vertical, black horizontal line at zero are considered significant ($p_{bootstrap}<0.05$). Solid lines represent training data, while the dotted lines represent test data. The solid box indicates the canonical correlation value (r). Components 1 to 6 correspond to components with excitability and impulsivity, depressive mood and distress, emotional and behavioral dysregulation, stress pathology, eating pathology, and social fear and avoidance symptoms. Notably, the green lines correspond to the same results as in Figure 4.

© 2025 Lett TA et al. *JAMA Psychiatry.*

Supplementary References

1. Lett TA, Vogel BO, Ripke S, et al. Cortical Surfaces Mediate the Relationship Between Polygenic Scores for Intelligence and General Intelligence. *Cereb Cortex*. 2020;30(4):2707-2718.

2. Schumann G, Loth E, Banaschewski T, et al. The IMAGEN study: reinforcement-related behaviour in normal brain function and psychopathology. *Mol Psychiatry*. 2010;15(12):1128-1139.

3. Goodman R, Ford T, Richards H, Gatward R, Meltzer H. The Development and Well-Being Assessment: description and initial validation of an integrated assessment of child and adolescent psychopathology. *J Child Psychol Psychiatry*. 2000;41(5):645-655.

4. Goodman R. The Strengths and Difficulties Questionnaire: a research note. *J Child Psychol Psychiatry*. 1997;38(5):581-586.

5. Saunders JB, Aasland OG, Babor TF, de la Fuente JR, Grant M. Development of the Alcohol Use Disorders Identification Test (AUDIT): WHO collaborative project on early detection of persons with harmful alcohol consumption--II. *Addiction*. 1993;88(6):791-804.

6. Vulser H, Lemaitre H, Artiges E, et al. Subthreshold depression and regional brain volumes in young community adolescents. *J Am Acad Child Adolesc Psychiatry*. 2015;54(10):832-840.

7. Ing A, Sämann PG, Chu C, et al. Identification of neurobehavioural symptom groups based on shared brain mechanisms. *Nat Hum Behav*. 2019;3(12):1306-1318.

8. Jia T, Ing A, Quinlan EB, et al. Neurobehavioural characterisation and stratification of reinforcement-related behaviour. *Nat Hum Behav*. 2020;4(5):544-558.

9. Fischl B, Dale AM. Measuring the thickness of the human cerebral cortex from magnetic resonance images. *Proc Natl Acad Sci U S A*. 2000;97(20):11050-11055.

10. Fischl B, Liu A, Dale AM. Automated manifold surgery: constructing geometrically accurate and topologically correct models of the human cerebral cortex. *IEEE Trans Med Imaging*. 2001;20(1):70-80.

11. Fischl B, Salat DH, van der Kouwe AJW, et al. Sequence-independent segmentation of magnetic resonance images. *Neuroimage*. 2004;23 Suppl 1:S69-84.

12. Fischl B, Sereno MI, Dale AM. Cortical surface-based analysis. II: Inflation, flattening, and a surface-based coordinate system. *Neuroimage*. 1999;9(2):195-207.

13. Fischl B, Sereno MI, Tootell RBH, Dale AM. High-resolution intersubject averaging and a coordinate system for the cortical surface. *Hum Brain Mapp*. 1999;8(4):272-284.

14. Wang Y, Shen Y, Liu D, et al. Evaluations of diffusion tensor image registration based on fiber tractography. *Biomed Eng Online*. 2017;16(1):9.

15. Bach M, Laun FB, Leemans A, et al. Methodological considerations on tract-based spatial statistics (TBSS). *Neuroimage*. 2014;100:358-369.

16. Zhang H, Yushkevich PA, Alexander DC, Gee JC. Deformable registration of diffusion tensor MR images with explicit orientation optimization. *Med Image Anal*. 2006;10(5):764-785.

17. Keihaninejad S, Zhang H, Ryan NS, et al. An unbiased longitudinal analysis framework for tracking white matter changes using diffusion tensor imaging with application to Alzheimer’s disease. *Neuroimage*. 2013;72:153-163.

18. Zhang S, Arfanakis K. Evaluation of standardized and study-specific diffusion tensor imaging templates of the adult human brain: Template characteristics, spatial normalization accuracy, and detection of small inter-group

© 2025 Lett TA et al. *JAMA Psychiatry.*

FA differences. *Neuroimage*. 2018;172:40-50.

19. Smith SM, Jenkinson M, Johansen-Berg H, et al. Tract-based spatial statistics: voxelwise analysis of multi-subject diffusion data. *Neuroimage*. 2006;31(4):1487-1505.

20. Knutson B, Fong GW, Adams CM, Varner JL, Hommer D. Dissociation of reward anticipation and outcome with event-related fMRI. *Neuroreport*. 2001;12(17):3683-3687.

21. Grosbras MH, Paus T. Brain networks involved in viewing angry hands or faces. *Cereb Cortex*. 2006;16(8):1087-1096.

22. Aron AR, Poldrack RA. The cognitive neuroscience of response inhibition: relevance for genetic research in attention-deficit/hyperactivity disorder. *Biol Psychiatry*. 2005;57(11):1285-1292.

23. Jenkinson M, Bannister P, Brady M, Smith S. Improved optimization for the robust and accurate linear registration and motion correction of brain images. *Neuroimage*. 2002;17(2):825-841.

24. Pruim RHR, Mennes M, van Rooij D, Llera A, Buitelaar JK, Beckmann CF. ICA-AROMA: A robust ICA-based strategy for removing motion artifacts from fMRI data. *Neuroimage*. 2015;112:267-277.

25. Greve DN, Fischl B. Accurate and robust brain image alignment using boundary-based registration. *Neuroimage*. 2009;48(1):63-72.

26. Avants BB, Tustison NJ, Song G, Cook PA, Klein A, Gee JC. A reproducible evaluation of ANTs similarity metric performance in brain image registration. *Neuroimage*. 2011;54(3):2033-2044.

27. Smith SM, Nichols TE, Vidaurre D, et al. A positive-negative mode of population covariation links brain connectivity, demographics and behavior. *Nat Neurosci*. 2015;18(11):1565-1567.

28. Shirer WR, Ryali S, Rykhlevskaia E, Menon V, Greicius MD. Decoding subject-driven cognitive states with whole-brain connectivity patterns. *Cereb Cortex*. 2012;22(1):158-165.

29. Mori S, Oishi K, Jiang H, et al. Stereotaxic white matter atlas based on diffusion tensor imaging in an ICBM template. *Neuroimage*. 2008;40(2):570-582.

30. Glasser MF, Coalson TS, Robinson EC, et al. A multi-modal parcellation of human cerebral cortex. *Nature*. 2016;536(7615):171-178.

31. Johnson SC. Hierarchical clustering schemes. *Psychometrika*. 1967;32(3):241-254.

32. Ward JH. Hierarchical Grouping to Optimize an Objective Function. *J Am Stat Assoc*. 1963;58(301):236-244.

33. Thirion B, Varoquaux G, Dohmatob E, Poline JB. Which fMRI clustering gives good brain parcellations? *Front Neurosci*. 2014;8:167.

34. Sen PK. Estimates of the Regression Coefficient Based on Kendall's Tau. *J Am Stat Assoc*. 1968;63(324):1379-1389.

35. Thiel H. A rank-invariant method of linear and polynomial regression analysis, Part 3. *Proceedings of Koninalijke*.

36. Tenenhaus M, Tenenhaus A, Groenen PJF. Regularized Generalized Canonical Correlation Analysis: A Framework for Sequential Multiblock Component Methods. *Psychometrika*. Published online May 23, 2017. doi:10.1007/s11336-017-9573-x

37. Tenenhaus A, Philippe C, Guillemot V, Le Cao KA, Grill J, Frouin V. Variable selection for generalized canonical correlation analysis. *Biostatistics*. 2014;15(3):569-583.

© 2025 Lett TA et al. *JAMA Psychiatry.*

38. Van de Geer JP. Linear relations amongk sets of variables. *Psychometrika*. 1984;49(1):79-94.

39. Witten DM, Tibshirani R, Hastie T. A penalized matrix decomposition, with applications to sparse principal components and canonical correlation analysis. *Biostatistics*. 2009;10(3):515-534.

40. Shah RD, Samworth RJ. Variable Selection with Error Control: Another Look at Stability Selection. *J R Stat Soc Series B Stat Methodol*. 2012;75(1):55-80.

41. Benjamini Y, Hochberg Y. Controlling the False Discovery Rate: A Practical and Powerful Approach to Multiple Testing. *J R Stat Soc Series B Stat Methodol*. 1995;57(1):289-300.

© 2025 Lett TA et al. *JAMA Psychiatry.*
