# Supplementary material for: Framework for Brain-Derived Dimensions of Psychopathology
Source: JAMA Psychiatry. 2025 Jun 18;82(8):778–89. doi: 10.1001/jamapsychiatry.2025.1246 (PMC12177734; doi:10.1001/jamapsychiatry.2025.1246)
Supplement: Supplement 2. — environMENTAL Consortium members [file jamapsychiatry-e251246-s002.pdf]

Supplemental Online Content: Nonauthor Collaborators
*First name, last name, and suffix (if applicable) are required and will appear in PubMed.

*Group Name(s): environMENTAL Consortium							
***First Name and Middle Initial(s)**	***Last Name**	***Suffix (eg, Jr, III)**	Academic Degrees	Institution	Location (city, state/province, country)	Role or Contribution, eg, chair, principal investigator	Group (if more than 1 Group listed in the byline) and/or Subgroup (eg, Steering Committee)
Gunter	Schumann			Centre for Population Neuroscience and Stratified Medicine, Department of Psychiatry and Neuroscience; Department of Psychiaty and Psychotherapy, Charité Universitätsmedizin, Berlin, Germany	Berlin, Germany		
Esther	Hitchen			Centre for Population Neuroscience and Stratified Medicine, Department of Psychiatry and Neuroscience; Department of Psychiaty and Psychotherapy, Charité Universitätsmedizin, Berlin, Germany	Berlin, Germany		
Elli	Polemiti			Centre for Population Neuroscience and Stratified Medicine, Department of Psychiatry and Neuroscience; Department of Psychiaty and Psychotherapy, Charité Universitätsmedizin, Berlin, Germany	Berlin, Germany		
Hedi	Kebir			Centre for Population Neuroscience and Stratified Medicine, Department of Psychiatry and Neuroscience; Department of Psychiaty and Psychotherapy, Charité Universitätsmedizin, Berlin, Germany	Berlin, Germany		
Tristram A.	Lett			Centre for Population Neuroscience and Stratified Medicine, Department of Psychiatry and Neuroscience; Department of Psychiaty and Psychotherapy, Charité Universitätsmedizin, Berlin, Germany	Berlin, Germany		
Nilakshi	Vaidya			Centre for Population Neuroscience and Stratified Medicine, Department of Psychiatry and Neuroscience; Department of Psychiaty and Psychotherapy, Charité Universitätsmedizin, Berlin, Germany	Berlin, Germany		

*First name, last name, and suffix (if applicable) are required and will appear in PubMed.

*First Name and Middle Initial(s)	*Last Name	*Suffix (eg, Jr, III)	Academic Degrees	Institution	Location (city, state/province, country)	Role or Contribution, eg, chair, principal investigator	Group (if more than 1 Group listed in the byline) and/or Subgroup (eg, Steering Committee)
Jean-Charles	Roy			Centre for Population Neuroscience and Stratified Medicine, Department of Psychiatry and Neuroscience; Department of Psychiaty and Psychotherapy, Charité Universitätsmedizin, Berlin, Germany	Berlin, Germany		
Henrik	Walter			1 - Department of Psychiatry and Psychotherapy, Charité Universitätsmedizin, corporate member of Freie Universität Berlin and Humboldt-Universität zu Berlin, Berlin, Germany 2 - German Center for Mental Health (DZPG), University Berlin-Potsdam, Germany	Berlin, Germany		
Andreas	Heinz			1 - Department of Psychiatry and Psychotherapy, Charité Universitätsmedizin, corporate member of Freie Universität Berlin and Humboldt-Universität zu Berlin, Berlin, Germany 2 - German Center for Mental Health (DZPG), University Berlin-Potsdam, Germany	Berlin, Germany		
Markus	Ralser			1 - Dept. of Biochemistry, Charité – Universitätsmedizin Berlin, Berlin, Germany 2 - Nuffield Department of Medicine, University of Oxford, UK 3 - Max Planck Institute for Molecular Genetics, Berlin, Germany	Berlin, Germany		
Sven	Twardziok			Berlin Institute of Health, Charité Universitätsmedizin, Berlin, Germany	Berlin, Germany		
Emin	Serin			Department of Psychiatry and Psychotherapy, Charité Universitätsmedizin, Berlin, Germany	Berlin, Germany		
Roland	Eils			Berlin Institute of Health, Charité Universitätsmedizin, Berlin, Germany	Berlin, Germany		
Marcel	Jentsch			Berlin Institute of Health, Charité Universitätsmedizin, Berlin, Germany	Berlin, Germany		
Ulrike	Taron			Berlin Institute of Health, Charité Universitätsmedizin, Berlin, Germany	Berlin, Germany		
Tatjana	Schütz			Berlin Institute of Health, Charité Universitätsmedizin, Berlin, Germany	Berlin, Germany		

Supplemental Online Content: Nonauthor Collaborators
*First name, last name, and suffix (if applicable) are required and will appear in PubMed.

***First Name and Middle Initial(s)**	***Last Name**	***Suffix (eg, Jr, III)**	Academic Degrees	Institution	Location (city, state/province, country)	Role or Contribution, eg, chair, principal investigator	Group (if more than 1 Group listed in the byline) and/or Subgroup (eg, Steering Committee)
Kerstin	Schepanski			Institute of Meteorology, Freie Universität Berlin, Berlin, Germany	Berlin, Germany		
Tobias	Banaschewski			Dept. of Child and Adolescent Psychiatry and Psychotherapy, Central Institute of Mental Health, German Center for Mental Health (DZPG), partner site Mannheim-Heidelberg-Ulm, Mannheim, Germany	Mannheim, Germany		
Maja	Neidhart			1 - Dept. of Psychiatry and Psychotherapy, CCM, Charite Universitaetsmedizin Berlin<br>2 - Dept. of Child and Adolescent Psychiatry and Psychotherapy, Central Institute of Mental Health, Medical Faculty Mannheim, Heidelberg University, Central Institute of Mental Health, Mannheim, Germany	Mannheim, Germany		
Andreas	Meyer-Lindenberg			Dept. of Psychiatry and Psychotherapy, Central Institute of Mental Health, German Center for Mental Health (DZPG), partner site Mannheim-Heidelberg-Ulm, Mannheim, Germany	Mannheim, Germany		
Heike	Tost			Dept. of Psychiatry and Psychotherapy, Central Institute of Mental Health, German Center for Mental Health (DZPG), partner site Mannheim-Heidelberg-Ulm, Mannheim, Germany,	Mannheim, Germany		
Nathalie	Holz			Dept. of Child and Adolescent Psychiatry and Psychotherapy, Central Institute of Mental Health, Mannheim, Germany, German Center for Mental Health (DZPG), partner site Mannheim-Heidelberg-Ulm, Germany	Mannheim, Germany		

Supplemental Online Content: Nonauthor Collaborators
*First name, last name, and suffix (if applicable) are required and will appear in PubMed.

***First Name and Middle Initial(s)**	***Last Name**	***Suffix (eg, Jr, III)**	Academic Degrees	Institution	Location (city, state/province, country)	Role or Contribution, eg, chair, principal investigator	Group (if more than 1 Group listed in the byline) and/or Subgroup (eg, Steering Committee)
Emanuel	Schwarz			1. Hector Institute for Artificial Intelligence in Psychiatry, Central Institute of Mental Health, Medical Faculty Mannheim, Heidelberg University, Mannheim, Germany<br>2. Department of Psychiatry and Psychotherapy, Central Institute of Mental Health, Medical Faculty Mannheim, Heidelberg University, Mannheim, Germany<br>3. German Center for Mental Health (DZPG), partner site Mannheim-Heidelberg-Ulm, Germany			
Argyris	Stringaris			Dept. of Child and Adolescent Psychiatry and Psychotherapy, Central Institute of Mental Health, Mannheim, Germany	Mannheim, Germany		
Nina	Christmann			Dept. of Child and Adolescent Psychiatry and Psychotherapy, Central Institute of Mental Health, Mannheim, Germany	Mannheim, Germany		
Karina	Janson			Dept. of Child and Adolescent Psychiatry and Psychotherapy, Central Institute of Mental Health, Mannheim, Germany	Mannheim, Germany		
Frauke	Nees			Institute of Medical Psychology and Medical Sociology, University Medical Center Schleswig-Holstein, Kiel, Germany	Kiel, Germany		
Maja	Neidhart			Institute of Medical Psychology and Medical Sociology, University Medical Center Schleswig-Holstein, Kiel, Germany	Kiel, Germany		
Beke	Seefried			Institute of Medical Psychology and Medical Sociology, University Medical Center Schleswig-Holstein, Kiel, Germany	Kiel, Germany		
Rieke	Aden			Institute of Medical Psychology and Medical Sociology, University Medical Center Schleswig-Holstein, Kiel, Germany	Kiel, Germany		
Karina	Janson			Institute of Medical Psychology and Medical Sociology, University Medical Center Schleswig-Holstein, Kiel, Germany	Kiel, Germany		

Supplemental Online Content: Nonauthor Collaborators
*First name, last name, and suffix (if applicable) are required and will appear in PubMed.

*First Name and Middle Initial(s)	*Last Name	*Suffix (eg, Jr, III)	Academic Degrees	Institution	Location (city, state/province, country)	Role or Contribution, eg, chair, principal investigator	Group (if more than 1 Group listed in the byline) and/or Subgroup (eg, Steering Committee)
Ole	Andreassen			Centre for Precision Psychiatry, Division of Mental Health and Addiction, Oslo University Hospital & Institute of Clinical Medicine, University of Oslo, , Oslo, Norway	Oslo, Norway		
Lars	Westlye			1 - Department of Psychology, University of Oslo, 2 - Centre for Precision Psychiatry, Division of Mental Health and Addiction, Oslo University Hospital & Institute of Clinical Medicine, University of Oslo, , Oslo, Norway	Oslo, Norway		
Dennis	van der Meer			Centre for Precision Psychiatry, Division of Mental Health and Addiction, Oslo University Hospital & Institute of Clinical Medicine, University of Oslo, , Oslo, Norway	Oslo, Norway		
Sara	Fernández-Cabello			1 - Department of Psychology, University of Oslo, 2 - Centre for Precision Psychiatry, Division of Mental Health and Addiction, Oslo University Hospital & Institute of Clinical Medicine, University of Oslo, , Oslo, Norway	Oslo, Norway		
Rikka	Kjelkenes			1 - Department of Psychology, University of Oslo, 2 - Centre for Precision Psychiatry, Division of Mental Health and Addiction, Oslo University Hospital & Institute of Clinical Medicine, University of Oslo, , Oslo, Norway	Oslo, Norway		
Helga	Ask			1 - Department of Psychology, University of Oslo, 2 - PsychGen Centre for genetic epidemiology and mental health, Norwegian Institute of Public Health, Oslo, Norway	Oslo, Norway		
Michael	Rapp			Social and Preventive Medicine, University of Potsdam, Potsdam, Germany	Potsdam, Germany		
Mira	Tschorn			Social and Preventive Medicine, University of Potsdam, Potsdam, Germany	Potsdam, Germany		
Sarah	Böttger			Social and Preventive Medicine, University of Potsdam, Potsdam, Germany	Potsdam, Germany		
Andre	Marquand			Cogntive Neuroscience, Radboud University Medical Centre, Nijmegen, The Netherlands	Nijmegen, The Netherlands		

*First name, last name, and suffix (if applicable) are required and will appear in PubMed.

***First Name and Middle Initial(s)**	***Last Name**	***Suffix (eg, Jr, III)**	Academic Degrees	Institution	Location (city, state/province, country)	Role or Contribution, eg, chair, principal investigator	Group (if more than 1 Group listed in the byline) and/or Subgroup (eg, Steering Committee)
Antoine	Bernas			Cogntive Neuroscience, Radboud University Medical Centre, Nijmegen, The Netherlands	Nijmegen, The Netherlands		
Gaia	Novarino			None, Institute of Science and Technology Austria, Vienna, Austria	Vienna, Austria		
Mel	Slater			Departament de Psicologia Clínica i Psicobiologia, Event Lab, Institute of Neuroscience of the University of Barcelona, Barcelona, Spain	Barcelona, Spain		
Jaime	Gallego			Departament de Psicologia Clínica i Psicobiologia, Event Lab, University of Barcelona, Barcelona, Spain	Barcelona, Spain		
Álvaro	Pastor			Departament de Psicologia Clínica i Psicobiologia, Event Lab, University of Barcelona, Barcelona, Spain	Barcelona, Spain		
Guillem	Feixas			Departament de Psicologia Clínica i Psicobiologia, Event Lab, University of Barcelona, Barcelona, Spain	Barcelona, Spain		
Francisco José	Eiroa-Orosa			Departament de Psicologia Clínica i Psicobiologia, Event Lab, University of Barcelona, Barcelona, Spain	Barcelona, Spain		
Markus	Nöthen			Institute of Human Genetics, University of Bonn School of Medicine and University Hospital Bonn, Bonn, Germany	Bonn, Germany		
Andreas	Forstner			Institute of Human Genetics, University of Bonn School of Medicine and University Hospital Bonn, Bonn, Germany	Bonn, Germany		
Isabelle	Claus			Institute of Human Genetics, University of Bonn School of Medicine and University Hospital Bonn, Bonn, Germany	Bonn, Germany		
Carina	Mathey			Institute of Human Genetics, University of Bonn School of Medicine and University Hospital Bonn, Bonn, Germany	Bonn, Germany		
Stefanie	Heilmann-Heimbach			Institute of Human Genetics, University of Bonn School of Medicine and University Hospital Bonn, Bonn, Germany , Life and Brain GmbH, Bonn, Germany	Bonn, Germany		

Supplemental Online Content: Nonauthor Collaborators
*First name, last name, and suffix (if applicable) are required and will appear in PubMed.

***First Name and Middle Initial(s)**	***Last Name**	***Suffix (eg, Jr, III)**	Academic Degrees	Institution	Location (city, state/province, country)	Role or Contribution, eg, chair, principal investigator	Group (if more than 1 Group listed in the byline) and/or Subgroup (eg, Steering Committee)
Per	Hoffmann			Institute of Human Genetics, University of Bonn School of Medicine and University Hospital Bonn, Bonn, Germany , Life and Brain GmbH, Bonn, Germany	Bonn, Germany		
Abigail	Miller			Institute of Human Genetics, University of Bonn School of Medicine and University Hospital Bonn, Bonn, Germany , Life and Brain GmbH, Bonn, Germany	Bonn, Germany		
Peter	Sommer			Not applicable, Ksilink, Strasbourg, France	Strasbourg, France		
Karen	Schmitt			Not applicable, Ksilink, Strasbourg, France	Strasbourg, France		
Johannes	Wilbertz			Not applicable, Ksilink, Strasbourg, France	Strasbourg, France		
Myrto	Patraskaki			Not applicable, Ksilink, Strasbourg, France	Strasbourg, France		
Viktor	Jirsa			, Aix-Marseille Université, Marseille, France	Marseille, France		
Spase	Petkoski			, Aix-Marseille Université, Marseille, France	Marseille, France		
Anastasios Polykarpos	Athanasiadis			, Aix-Marseille Université, Marseille, France	Marseille, France		
Bernhard	Spanlang			, Virtual Bodyworks, Barcelona, Spain	Barcelona, Spain		
Charlie	Pearmund			, Virtual Bodyworks, Barcelona, Spain	Barcelona, Spain		
Sören	Hese			Department of Earth Observation, Friedrich Schiller University Jena, Jena, Germany	Jena, Germany		
Paul	Renner			1 - Department of Earth Observation, Friedrich Schiller University Jena, Jena, Germany<br>2 - Institute of Meteorology, Freie Universität Berlin, Berlin, Germany	1 - Jena, Germany<br>2 - Berlin, Germany		
Tianye	Jia			Institute of Science and Technology for Brain-Inspired Intelligence, Fudan University, Fudan, China	Fudan, China		
Xiao	Chang			Institute of Science and Technology for Brain-Inspired Intelligence, Fudan University, Fudan, China	Fudan, China		
Jiacan	Yuan			, Fudan University, Fudan, China	Fudan, China		
Yuxiang	Dai			, Fudan University, Fudan, China	Fudan, China		
Yunman	Xia			, Fudan University, Fudan, China	Fudan, China		
Yuzhu	Li			, Fudan University, Fudan, China	Fudan, China		
Yanqing	Zhang			, Fudan University, Fudan, China	Fudan, China		

Supplemental Online Content: Nonauthor Collaborators
*First name, last name, and suffix (if applicable) are required and will appear in PubMed.

***First Name and Middle Initial(s)**	***Last Name**	***Suffix (eg, Jr, III)**	Academic Degrees	Institution	Location (city, state/province, country)	Role or Contribution, eg, chair, principal investigator	Group (if more than 1 Group listed in the byline) and/or Subgroup (eg, Steering Committee)
Vince	Calhoun			, Tri-institutional Center for Translational Research in Neuroimaging and Data Science, Georgia State University, Georgia Institute of Technology, and Emory University, Atlanta, GA, USA., Atlanta, USA	Atlanta, USA		
Paul	Thompson			Stevens Neuroimaging & Informatics Institute, Keck School of Medicine, University of Southern California, Los Angeles, USA	Los Angeles, USA		
Nicholas	Clinton			Geo, Google LLC, Mountain View, CA, USA	Mountain View, CA, USA		
Sylvane	Desrivières			Social, Genetic and Developmental Psychiatry Centre, King's College London, London, United Kingdom	London, United Kingdom		
Kofoworola	Agunbiade			Social, Genetic and Developmental Psychiatry Centre, King's College London, London, United Kingdom	London, United Kingdom		
Xinyang	Yu			Social, Genetic and Developmental Psychiatry Centre, King's College London, London, United Kingdom	London, United Kingdom		
Zuo	Zhang			1 - Social, Genetic and Developmental Psychiatry Centre, King's College London, London, United Kingdom<br>2 - Institute for Mental Health, School of Psychology, University of Birmingham, Birmingham, United Kingdom	1 - London, United Kingdom<br>2 - Birmingham, United Kingdom		
Di	Chen			Social, Genetic and Developmental Psychiatry Centre, King's College London, London, United Kingdom	London, United Kingdom		
Allan	Young			Psychological Medicine, King's College London, South London & Maudsely NHS Foundation Trust, London United Kingdom	London, United Kingdom		
Ameli	Schwalber			concentris research mamagement gmbh, Fürstenfeldbruck, Germany	Fürstenfeldbruck, Germany		
Vanessa	Köhler			concentris research mamagement gmbh, Fürstenfeldbruck, Germany	Fürstenfeldbruck, Germany		
Bernd	Stahl			School of Computer Science, University of Nottingham, Nottingham, United Kingdom	Nottingham, United Kingdom		

Supplemental Online Content: Nonauthor Collaborators
*First name, last name, and suffix (if applicable) are required and will appear in PubMed.

***First Name and Middle Initial(s)**	***Last Name**	***Suffix (eg, Jr, III)**	Academic Degrees	Institution	Location (city, state/province, country)	Role or Contribution, eg, chair, principal investigator	Group (if more than 1 Group listed in the byline) and/or Subgroup (eg, Steering Committee)
George	Ogoh			School of Computer Science, University of Nottingham, Nottingham, United Kingdom	Nottingham, United Kingdom		
Tamara	Schikowski			, IUF Leibniz Research Institute for Environmental Medicine, Düsseldorf, Germany	Düsseldorf, Germany		
Ragnhild	Brandlistuen			The Norwegian Mother, Father and Child Cohort Study, Norwegian Institute of Public Health, Oslo, Norway	Oslo, Norway		
