# Supplementary material for: Framework for Brain-Derived Dimensions of Psychopathology
Source: JAMA Psychiatry. 2025 Jun 18;82(8):778–89. doi: 10.1001/jamapsychiatry.2025.1246 (PMC12177734; doi:10.1001/jamapsychiatry.2025.1246)
Supplement: Supplement 3. — Data sharing statement [file jamapsychiatry-e251246-s003.pdf]

Data Sharing Statement

Lett. Framework for Brain-Derived Dimensions of Psychopathology. *JAMA Psychiatry*. Published June 18, 2025. doi:10.1001/jamapsychiatry.2025.1246

Data

Data available: Yes
Data types: Deidentified participant data
How to access data: IMAGEN data are available from a dedicated database at https://imagen-project.org. STRATIFY/ESTRA data are available from the IMAGEN database at https://stratify-project.org. The SGCCA statistical model, which includes aggregated data, will be shared upon reasonable request to the corresponding authors at tristram.lett@charite.de or gunter.schumann@charite.de.
When available: With publication

Supporting Documents

Document types: Statistical/analytic code
How to access documents: https://github.com/trislett/sgcca-psychiatry-nosology.git
When available: With publication

Additional Information

Who can access the data: Anyone requesting the data.
Types of analyses: The SGCCA statistical model, which includes aggregated data, will be shared upon reasonable request to the corresponding authors at tristram.lett@charite.de or gunter.schumann@charite.de.
Mechanisms of data availability: Pseudo-anonymised IMAGEN data are available from a dedicated database at https://imagen-project.org, and STRATIFY/ESTRA data are available from the IMAGEN database at https://stratify-project.org after approval of a proposal.
